# Effective normalization for copy number variation in Hi-C data

**DOI:** 10.1186/s12859-018-2256-5

**Published:** 2018-09-06

**Authors:** Nicolas Servant, Nelle Varoquaux, Edith Heard, Emmanuel Barillot, Jean-Philippe Vert

**Affiliations:** 1 Institut Curie, PSL Research University, Paris, F-75005 France; 2 INSERM, U900, Paris, F-75005 France; 3 Mines ParisTech, PSL Research University, CBIO-Centre for Computational Biology, Paris, F-75006 France; 4 Department of Statistics, University of California, Berkeley, USA; 5 Berkeley Institute for Data Science, Berkeley, USA; 6 Institut Curie, PSL Research University, CNRS UMR3215, INSERM U934, Paris, F-75005 France; 7 Ecole Normale Supérieure, PSL Research University, Department of Mathematics and Applications, Paris, F-75005 France

**Keywords:** Normalization, Hi-C, Cancer, Copy-number

## Abstract

**Background:**

Normalization is essential to ensure accurate analysis and proper interpretation of sequencing data, and chromosome conformation capture data such as Hi-C have particular challenges. Although several methods have been proposed, the most widely used type of normalization of Hi-C data usually casts estimation of unwanted effects as a matrix balancing problem, relying on the assumption that all genomic regions interact equally with each other.

**Results:**

In order to explore the effect of copy-number variations on Hi-C data normalization, we first propose a simulation model that predict the effects of large copy-number changes on a diploid Hi-C contact map. We then show that the standard approaches relying on equal visibility fail to correct for unwanted effects in the presence of copy-number variations. We thus propose a simple extension to matrix balancing methods that model these effects. Our approach can either retain the copy-number variation effects (LOIC) or remove them (CAIC). We show that this leads to better downstream analysis of the three-dimensional organization of rearranged genomes.

**Conclusions:**

Taken together, our results highlight the importance of using dedicated methods for the analysis of Hi-C cancer data. Both CAIC and LOIC methods perform well on simulated and real Hi-C data sets, each fulfilling different needs.

**Electronic supplementary material:**

The online version of this article (10.1186/s12859-018-2256-5) contains supplementary material, which is available to authorized users.

## Background

The spatial organization of the genome and the physical interactions occurring within and between chromosomes can play important roles in gene regulation and in genome function in general. The organization and folding of mammalian chromosomes within the nucleus involve multiple hierarchical chromatin structures (see Bonev et al. [1] for a review). At the megabase-scale, the genome in the interphase nucleus is divided into compartments of open and closed chromatin, that are respectively associated with gene-rich, actively transcribed regions and gene-poor, silent regions [2, 3]. Compartment organization varies across physiological conditions and during cell differentiation [4, 5]. At the sub-megabase scale, chromosomes are also partitioned into topological associated domains (TADs). These have been proposed as possible functional units of regulation, and are generally preserved across cell types, as well as being conserved between mammals [3, 4, 6, 7]. TAD boundaries are frequently associated with CTCF binding sites. CTCF is also involved in the establishment of chromatin loops between convergent target sites [3]. These chromatin loops are believed to provide scaffold for promoter-enhancer contacts and can therefore be implicated in gene activation (see Bouwman et al. [8] for a review).

Given the important recent insights that chromosome conformation techniques have provided into 3D genome organization in a normal context, the application of such approaches to a disease context offers great promises to explore the effect of perturbations in 3D genomic organization on cell regulation (see Kirjger et al. [9] for a review). At a high enough resolution, such techniques can be used to characterize links between disease-associated sequence variants and the gene regulatory landscape. For example, structural variants can disrupt boundaries between TADs, and consequently can act as driver events in the mis-regulation of associated gene expression [10, 11].

Over the past decade, major advances have been made in both high-throughput sequencing techniques and data availability from large patient cohorts across multiple cancer types, enabling a comprehensive and systematic exploration of genomic and epigenomic landscapes of a wide variety of cancers. While cancer has been shown to have a genetic component, our appreciation of the inherent epigenetic complexity is more recent and has dramatically increased over the last few years. At the genetic level, cancer is frequently associated with the sequential acquisition of somatic variants, both at the single nucleotide and at the copy number levels [12]. The different alterations that characterize tumors are usually caused by a few functional driver events, which occur among many non-functional passenger events, mainly located in the non-coding part of the genome [13]. One of the exciting discoveries that has emerged from systematic sequencing of cancer genomes was the high frequency of mutations in genes known to regulate epigenetic processes such as chromatin associated proteins, DNA methylation, or histone variants and modifications [14]. The contribution of altered epigenomes in the process of tumorigenesis is thus at last being unraveled thanks to the combination of genomic and epigenomic interrogation. More recently, genetic and epigenetic alterations in the non-coding part of the genome, including distal regulatory elements such as enhancers or insulators, have been reported and found to impact gene expression in cancer [15]. This has led to intense interest in the spatial proximity and 3D organization of cancer genomes. Losada et al. [16] reviews the effect of somatic mutations in cohesin complex proteins (which play a citical role in TADs organization and chromosome looping) in various types of cancer. Groeschel et al. [17] and Taberlay et al. [18] describe how disruptions in genome organization (respectively in leukemia and prostate cancer) lead to major epigenetic and transcriptional changes. Lastly, Hnisz et al. [19], Weischenfeldt et al. [20], and Beroukhim et al. [21] show how disruptions in long range DNA looping and genome rearrangements lead to enhancer hijacking and Flavahan et al. [22] link insulator dysfunctions to oncogene activation in cancer. Thus, changes in chromosome conformation at different scales are now considered as key potential players in cancer, as well as important potential biomarkers.

Developing accurate and quantitative methods to analyze the chromatin conformation derived from disease-associated cells/tissues is therefore of increasing interest to a wide community of researchers and pathologists. In addition to standard microscopy approaches, several 3C-based methods are now used: these rely on digestion and religation of fixed chromatin to estimate the probability of contact between two genomic loci (see Ramani et al. [23] for a review). In Hi-C experiments, the contact frequencies between two genomic loci are roughly proportional to the reads counts observed between two regions after sequencing [2]. However, as is the case for many high-throughput technologies, the raw contact frequencies are affected by systematic biases such as GC content, mappability, or restriction fragment size [24]. Estimating and correcting these biases is therefore an important step in ensuring accurate downstream analysis. In the past few years, several methods and packages have been developed to normalize Hi-C data (see Ay and Noble [25] for a review). These methods fall into two main categories: explicit factor correction methods or matrix balancing algorithms. Explicit-factor normalization methods require an a priori knowledge of the Hi-C systematic biases. Yaffe et al. [24] first proposed a non-parametric model to estimate the probability of observing a contact between two loci given these biases. The main limitation of this method is its computational cost. Subsequently, Hu et al. [26] proposed a much faster explicit correction method, based on Poisson or Negative Binomial regression which can be applied at the bin resolution, and gives similar performance compared to the original method. Unlike the explicit factor correction methods, the matrix balancing methods do not assume any specific source of biases, and are in theory able to correct for all unwanted variances in the contact map [3, 27, 28]. Applying such methods leads to an optimization problem that can be solved efficiently and precisely using the Sinkhorn and Knopp algorithm [28], or the Knight and Ruiz algorithm [3].

In the context of cancer Hi-C data, an additional perturbation related to chromosomal rearrangements must be considered. Amplified genomic regions have a greater chance of being pull-down during the library preparation, while genomic regions with lower copy numbers are more difficult to detect. To date, such copy number variants (CNVs) are usually ignored in cancer Hi-C data normalization, although they raise interesting and important questions both at the biological and methodological levels. The real impact of CNVs on contact frequencies remains difficult to assess. For instance, a tandem amplification has a very different impact on local chromatin organization compared to the gain of a complete chromosome. Similarly, a genomic duplication could lead to different changes in contact frequencies depending on whether the event occurs within a TADs or across/at a TAD boundary [10]. Addressing the question of CNVs during normalization is therefore an important challenge in the analysis of Hi-C data and their interpretation in the context of genetic and epigenetic mis-regulation in disease.

The question of how copy number signal should be treated depends mainly on the related biological questions. One strategy is to consider the copy number effect as an unwanted effect, and to remove it during the normalization step [29]. This strategy indeed makes sense for the detection of a genome-wide list of significant contacts, or for the direct comparison of samples with different chromosomal rearrangement profiles. On the other hand, the signal from copy number alterations can also be considered as important biological information, that can be of interest for 3D modeling, genome reconstruction of cancer cells, or to simply further characterize the genomic landscape of a tumor [30].

Here, we propose to further explore the impact of CNVs on Hi-C data and provide tools that deal with its effects on data normalization. First, we develop a model simulating large copy number rearrangements on a diploid Hi-C contact map. Using such simulated data, we demonstrate that the naive matrix balancing algorithm which is commonly used to normalize Hi-C data, cannot be applied to cancer Hi-C data. We then propose two methods that extend the ICE algorithm and correct the data from systematic biases, either considering the CNVs as a bias to remove or as an interesting signal to conserve in the data structure. Finally, we apply these methods to several disease associated Hi-C data sets, demonstrating their relevance.

## Results

### Simulating the effect of copy number variations on Hi-C data

**Fig. 1:**
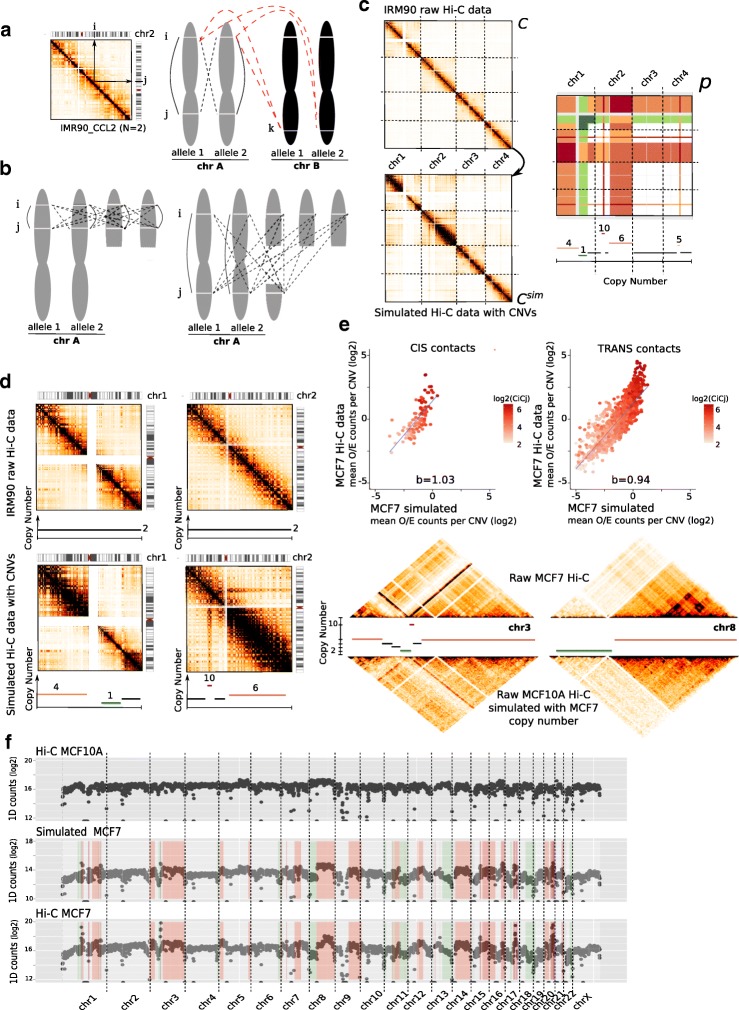
Simulation of cancer Hi-C data. **a**. In Hi-C data from diploid cells, the contact frequency measured between two loci *i* and *j* is equal to the sum of 2 cis interactions (black solid lines) occurring within an individual allele and of 2 trans interactions between homologous chromosomes (transH, black dashed lines). In addition, the contact frequency observed in trans between loci *i* and *k* is the sum of 4 interactions between non homologous chromosomes (red dashed lines). **b**. In the context of segmental rearrangement, these properties can be extended and generalized if loci *i* and *j* belong to the same DNA segment, or to different segments (see “Methods” section and Additional file 1: Figure S1). **c**. Simulation of cancer Hi-C data from normal diploid (C) data by calculating the scaling factor matrix (p). Colors in scaling factor matrix represent the level of gains (red) and loss (green) to simulate. For each interaction $C^{\text {sim}}_{ij}$, the simulated count is finally estimated using a binomial down-sampling method (see “Methods” section). **d**. Intra-chromosomal maps of chromosome 1 and 2 before (top) and after (bottom) simulation of copy number changes. Copy number effects are characterized by blocks of high/lower signal. Overall, the simulation conserves the structure and the counts/distance properties of the Hi-C maps. **e**. Validation of the simulation model using Hi-C data from MCF10A cell line from which we simulated the expected copy number of MCF7 cancer cell line (MCF7 simulated). The mean O/E (Observed/Expected) counts per block of copy number of intra (cis) and inter-chromosomal (trans) maps at 1 Mb resolution is represented. Looking at the intra-chromosomal maps of chr3 and 8 demonstrates that our model efficiently simulates large copy number events. **f**. 1D genome-wide profiles of near-diploid MCF10A, simulated MCF7 and real MCF7 Hi-C data. MCF7 gain and losses are represented in red and green

Due to the large number of genomic and epigenomic factors possibly involved, predicting the true effect of copy-number variations on the 3D organization of the genome is challenging. We propose a simple mathematical model to simulate the effect of abnormal karyotypes on a diploid Hi-C data set by estimating the enrichment in interactions due to CNVs (see “Methods” section and Fig. 1). Our model is based on the assumptions that (1) copies of chromosomes are independent and have similar 3D structure and (2) that the impact of copy number changes is higher than 3D structure variations that occur across cell types. Therefore, for a given copy number profile, our model will estimate the expected Hi-C contact maps in the presence of CNVs (Fig. 1d).

In order to validate our simulation model, we exploited available Hi-C data from two epithelial cell lines: the MCF7 breast cancer cell line and the MCF10A near-diploid, non-tumorigenic cell line [5]. We extracted the copy number information of the MCF7 line from Affymetrix SNP6.0 array, filtering out any altered segments that were lower than the MCF10A’s Hi-C map resolution (1 Mb) and applied our simulation model on the normal-like data, thus obtaining a simulation of MCF7’s abnormal Hi-C data. We then compared our simulated results with the real MCF7 Hi-C data set. As expected, the contact counts (for both the simulated data and the real data) are correlated with the copy number (Fig. 1e). Both our simulations and the real data show blocks of higher/lower contact frequencies in regions affected by large copy number variants. Interestingly, for the highest copy numbers, both profiles increase concurrently, but not at the same rate. One explanation would be that these regions of very high copy number correspond to complex rearrangements such as tandem focal amplifications in cis and translocation in trans. Their linear proximity on the genome would therefore explain the massive increase of contact frequencies that we observed in real data, and which are not modeled by our simulation. We then summarized both data in one dimension (1D) by summing the contact frequencies over each row. Overall, the simulated MCF7 profile is well correlated with the profile of real MCF7 Hi-C data (Spearman cor =0.877, Fig. 1f, Table S2). We can observe that the sum of interactions for a genomic window is proportional to the copy number. These observations lead us to believe that our simulation method appropriately models the effect of copy number variations in Hi-C data.

### The ICE normalization is not suitable for cancer Hi-C data

Several methods have been proposed to remove unwanted technical and biological variations from Hi-C data. Among them, the matrix-balancing methods leverage a small number of hypotheses on the biases and on the properties of Hi-C data to formulate their normalization procedure: these do not assume any specific source of bias, and are (as long as the hypotheses are fulfilled) able to correct for any factors affecting contact frequencies [27, 28]. In this context, the iterative correction method (ICE, [28]) has been successfully applied to many diploid Hi-C data sets. ICE relies on two assumptions: (1) the bias between two regions *i* and *j* can be represented as the product of individual biases of these regions : $N^{\text {ICE}}_{ij} = \beta _{i} \beta _{j} C_{ij}$; (2) each bin should interact approximately the same number of times: ${\sum \nolimits }_{i} N^{\text {ICE}}_{ij} =k$, where *C* represents the raw count matrix, *N*^ICE^ the ICE normalized count matrix, *β* the bias vectors and *k* a constant.

We therefore applied our simulation model to assess the ability of the ICE normalization method to correct for CNVs. We simulated two data sets with different properties from the publicly available human IMR90 Hi-C data [3]; a highly rearranged data set with segmental gains, losses and a focal amplification up to 10 copies (Fig. 1c) and a case of aneuploidy with gain or loss of entire chromosomes (Additional file 1: Figure S2a). While the simulations were performed genome-wide, we restricted the CNVs to the first chromosomes to ease the results interpretation and visualization. The ground-truth normalized data was found by applying ICE to the original diploid data. We were thus able to assess the performance of ICE to correct for unwanted sources of variation, including the copy number, by comparing the obtained matrices to the ground-truth.

**Fig. 2:**
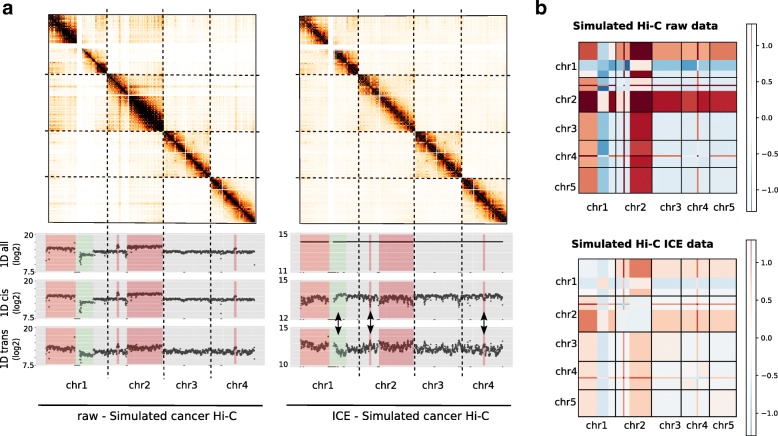
Impact of matrix balancing normalization on simulated cancer Hi-C data. **a**. Simulated Hi-C contact maps (500 kb resolution) of the first four chromosomes and contact frequencies presented as the sum of genome-wide contacts per locus, using either all (inter and intra-chromosomal), cis (intra-chromosomal) or trans (inter-chromosomal) contacts. Rearranged regions are highlighted in red (gain) or green (loss). The 1D profile of ICE data is constant genome-wide as expected under the assumption of equal visibility. However, the iterative correction on simulated cancer data results in an shift of contacts between altered regions (see arrows for examples). **b**. Block-average error matrix of simulated raw and ICE cancer data (150 Kb resolution) (See Additional file 1: Method 1.4). The iterative correction does not allow to correct for segmental copy number bias

Before running the ICE normalization, we first represented the data in 1D, by summing each row of the matrix. As previously mentioned, the sum of genome-wide interactions per bin is proportional to the copy number (Fig. 2a). After applying ICE, each genomic region now interacts the same number of times genome-wide, as expected. However, ICE leads to an imbalance between cis and trans contact counts; cis contact counts are now depleted for regions with high copy number, and trans contact counts are enriched (Fig. 2b). On the other hand, lost regions now present higher contact probabilities than regions of gain in cis. The same conclusions can be made in the context of aneuploidy (Additional file 1: Figure S2). However, we notice that in this case, ICE can yield to the expected results if the analysis is restricted to intra-chromosomal contacts.

If the downstream analysis is restricted to intra-chromosomal interactions, one may ask whether applying ICE independently to each intra-chromosomal maps could mitigate the introduction of biases. We therefore independently normalized by ICE all intra-chromosomal maps. Although the effects are less strong, we observed the same phenomenon in complex rearrangements (Additional file 1: Figure S3).

Altogether, these results demonstrate that ICE does not adequately normalize data from cells with abnormal karyotypes. More importantly, using ICE on cancer Hi-C data can lead to a misinterpretation of the contact probabilities between rearranged regions. Its use is therefore not recommended in this context.

### Estimation of copy number from Hi-C data

Analyzing cancer samples usually requires access to CNV profiles. External sources of data (such as whole-genome sequencing or microarray data) can be used to infer DNA breakpoints along the genome, and thus to define DNA segments of equal copy number. If such data is not available, we propose to directly infer the copy number profile from the Hi-C contact maps (see “Methods” section). Although in theory, all sequencing reads are useful to estimate the copy number profile, we first validated that using the counts from the contact maps (i.e. the subset of valid interaction products) is sufficient. For this, we compared our estimated copy number profile from MCF7 and T47D Hi-C contact maps, with the results of the Control-FREEC software [31] that directly uses the aligned sequencing reads to call the CNVs (Additional file 1: Figure S4). We observe a good correlation between both methods (MCF7 spearman cor =0.888, T47D spearman cor =0.855) therefore validating our approach.

**Fig. 3:**
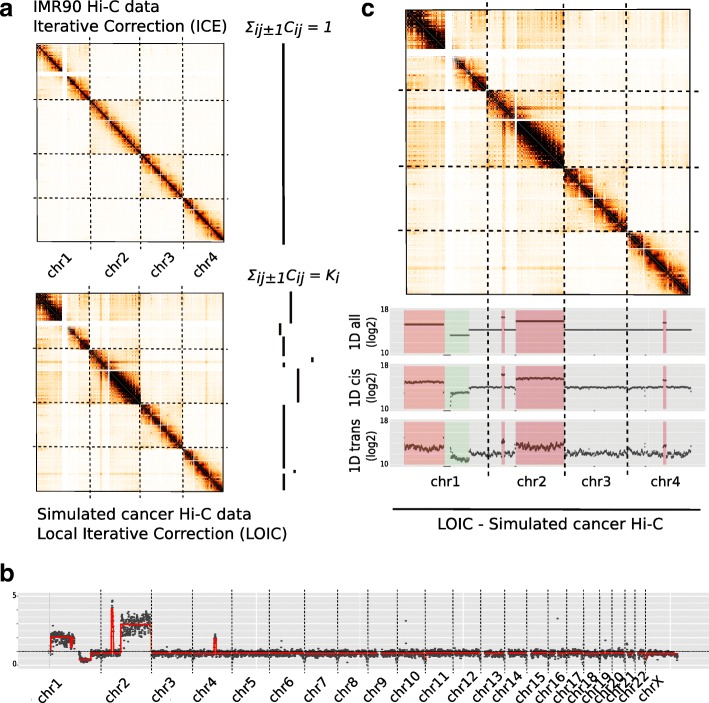
Generalization of matrix balancing algorithms for cancer Hi-C data. **a**. Rationale of LOIC method versus standard ICE method. The LOIC method extends the ICE normalization by constraining the genome-wide Hi-C 1D profile to follow the copy number signal. **b**. Segmentation of the Hi-C 1D genome-wide profile of simulated cancer data. The red line represents the smoothing line that estimate the copy number level. **c**. LOIC normalized Hi-C contact maps of simulated data on the first four chromosomes. The 1D profiles are represented by the sum of genome-wide contacts at each locus using either all (inter and intra-chromosomal), cis (intra-chromosomal) or trans (inter-chromosomal) contacts. As a results, we can see that the LOIC method allows to normalize cancer Hi-C data keeping into account the copy number information

We then applied our segmentation strategy to our Hi-C simulated data (Fig. 3b). In order to assess the robustness of this approach, we further simulated 100 additional data sets with distinct CNVs profiles. Results on this larger number of data sets show a 91% recall and a precision of 62.4%. In particular, we observe that the precision of breakpoint detection can sometimes be lower in highly amplified regions. Finally, we also applied our segmentation procedure to the IMR90 diploid data set. As expected, we obtain a nearly uniform copy number profile (Additional file 1: Figure S6c). Interestingly, we frequently observe a decrease of contacts at telomeric regions which therefore results in a breakpoint in the segmentation. This telomeric pattern is expected, even in a diploid sample, as the assumption of equal visibility in these regions is questionable.

### LOIC: a novel normalization strategy for cancer Hi-C data

As presented above, ICE relies on the assumption of equal visibility of each genomic bin. In the presence of copy number variations, this assumption does not hold: genomic bins with higher copy number variations will interact overall more frequently than genomic bins of lower CNVs. In addition, the copy number effect between loci *i* and *j* (*B*_*ij*_), cannot be decomposed into the product of an effect in locus *i* and an effect in locus *j*, thereby also violating the ICE hypothesis. Instead, we propose to extend the ICE model, through the assumption that equal visibility remains true across regions of identical copy number. In addition, biases associated to fragments (such as fragment length, GC-content, or mappability) can still be decomposed into the product of two region-specific biases.

We thus first propose to extend ICE by assuming that the sum of contacts for a given genomic bin is constant across genomic bins of identical copy number (Fig. 3a): ${\sum \nolimits }_{i} C^{LOIC}_{ij} = k_{j}$, where *k*_*j*_ is the interaction profile associated to the copy number of *j* (see “Methods” section). We refer to this method as a LOcal Iterative Correction (LOIC). When there is no copy number alteration, LOIC solves exactly the same problem as ICE.

We applied the LOIC procedure to our highly rearranged simulated Hi-C data set using the breakpoint positions estimated by our segmentation procedure (Fig. 3c). As expected, we observe that the genome-wide sum of contacts of each bin is proportional to the copy number, and that bins within a DNA segment are normalized to the same level of interactions. The previous effects on the cis and trans sum of contacts observed using the standard ICE strategy, is no longer found. In addition, we calculated the effective fragment length, the GC content and the mappability features for each 500 Kb bin as already proposed [26], and then represented the average contact frequencies among those genomic features. Despite a few local enrichments due to CNVs, we observe that LOIC normalization is as effective as ICE normalization for correction of GC content, effective fragment length and mappability (Additional file 1: Figure S5). We then turned to the aneuploid simulated data set. In this case, LOIC enables conservation of the inter-chromosomal scaling factor due to CNVs. Differences in intra-chromosomal maps between ICE and LOIC remain negligible and are related to the segmentation profile (Additional file 1: Figure S6a, b).

In conclusion, the LOIC strategy presented here can be seen as a generalization of the ICE method allowing to correct the Hi-C maps for systematic biases while keeping the copy number signal. In this sense, applying either methods to a diploid data set leads to identical results.

### CAIC: estimating and removing the copy-number effect on cancer Hi-C data

We also set out to estimate and to correct the effect introduced by copy number changes. We assume that copy number effects can be represented as a block-constant matrix where each block is delimited by a copy number change (see “Methods” section). In addition, we assume that, on average, each pair of loci interacts the same way as any other pair of loci at the same genomic distance *s*. In summary, the raw interaction count *C*_*ij*_ is roughly equal to the product of a CNV bias *B*_*ij*_ and the expected contact count at genomic distance *s*: *C*_*ij*_≃*B*_*ij*_*e*_*s*(*i*,*j*)_. We thus cast an optimization problem to find the CNV block biases *B* and the expected contact count at genomic distance *s* (see “Methods”section). We refer to this method as CNV-Adjusted Iterative Correction (CAIC).

**Fig. 4:**
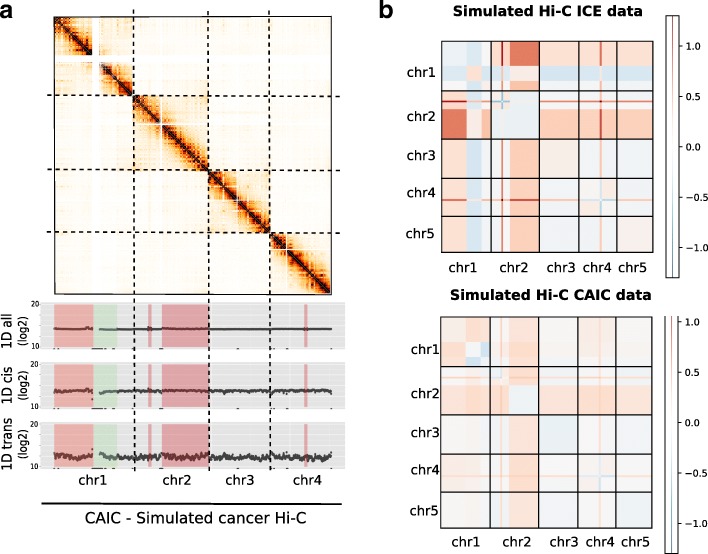
CNV-adjusted normalization of cancer Hi-C data. **a.** Hi-C contact maps of the four first chromosomes of our highly rearranged simulated data, together with the 1D signal of all, cis and trans data. Regions in red and green correspond to simulated gains and losses. **b.** Block-average error matrix of simulated ICE and CAIC Hi-C data. The CAIC efficiently removed the CNV effect, whereas the ICE normalization does not allow to correct for its effect

We applied CAIC normalization to the two simulated data sets. Looking at the 1D signal of the CAIC normalized data using the cis and trans data validates that the method tends to remove the CNV effect (Fig. 4a). In addition, the unbalanced effect that we previously observed with the ICE normalized data disappeared. We then divided the normalized contact matrices by the expected count matrices, thus removing the structure due to genomic proximity. Taking the average per block, we observe that the expected CAIC matrices are much more uniform than the ICE normalized matrices (Additional file 1: Figure S7 and S8). On the aneuploid simulated data set, it is worth noting that ICE and CAIC yield very close results in cis. We then compared the normalized contact maps to the ground-truth by computing the error matrix as well as three additional error measures (see Fig. 4b and Additional file 1: Methods 1.4). We observe that the copy number effect is well removed both on the aneuploid and highly rearranged data set (Table S3).

Altogether, these results demonstrate that the CAIC normalization procedure effectively removes copy-number effects from Hi-C contact maps.

### Application to breast cancer Hi-C data

**Fig. 5:**
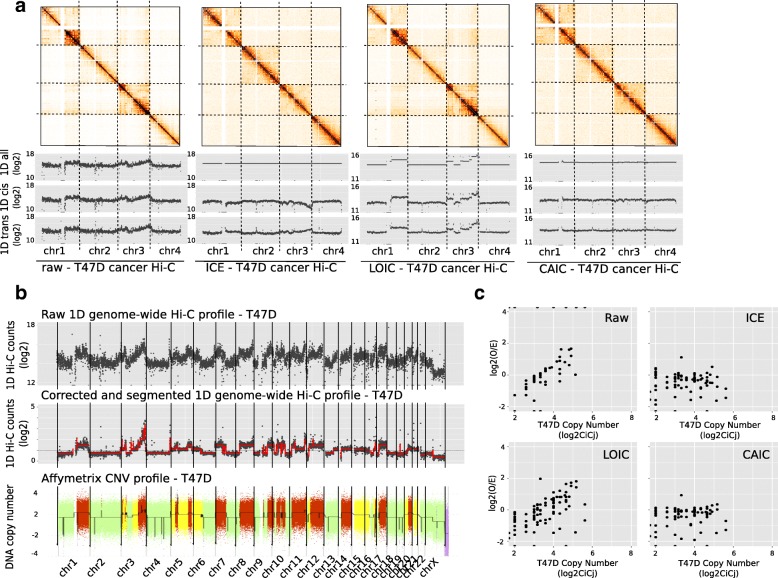
Normalization of T47D Hi-C data. **a**. Hi-C contact maps (250 Kb resolution) of the first four chromosomes of T47D cancer Hi-C sample. When looking at the 1D cis and trans profiles, we observed that ICE introduces a bias in the normalized data, therefore validating the observation made on the simulated data. We then applied the LOIC and CAIC normalizations in order to efficiently correct the data from systematic bias, while removing or keeping the CNVs effect. **b**. Estimatation of the copy number signal from the Hi-C data after correction and segmentation of the 1D profile. The inferred copy number signal from the Hi-C data are highly correlated with the copy number profile from Affymetrix SNP6.0 array. **c**. Correlation of raw and normalized contact frequencies with the copy number

A number of studies performed Hi-C experiments on cancer samples or cell lines [5, 18, 32]. We further explored the effect of our normalization procedures on two previously published Hi-C data from breast cancer cell lines: T47D [32] and MCF7 [5]. We processed the T47D and MCF7 samples from raw data files to raw contact maps using the HiC-Pro pipeline [33]. As already seen in our simulation data (Fig. 2a), we observe a strong copy number effect on the raw contact maps with respectively higher/lower contact frequency on gained/lost DNA regions in both samples (Fig. 5, Additional file 1: Figure S9). Applying ICE on these data sets does not entirely remove the copy number effect, and tends to flip the coverage profile between gained/lost regions in cis, therefore validating our previous observations on simulated data (Fig. 5a, Additional file 1: Figure S9a).

In order to estimate the copy number signal from these cell lines, we segmented the 1D Hi-C profile as previously described. Interestingly, on both T47D and MCF7 data we observed a very good correlation between the copy number signal extracted directly from the Hi-C data and the copy number profile extracted from SNP6 Affymetrix array (Fig. 5b, Additional file 1: Figure S9b, Spearman cor =0.87 for both MCF7 and T47D data) We then applied the LOIC strategy presented above, so that the sum of each column/row follows the segmentation profile extracted from the data. As expected, we observe that the LOIC normalized contact maps conserves the copy number properties, and that the biases introduced by the ICE normalization no longer hold true. In addition, we also applied the CAIC normalization to correct for CNVs signal. Looking at the correlation between Hi-C counts and the copy number signal validates the efficiency of the methods (Fig. 5c, Additional file 1: Figure S10). In conclusion, applying the LOIC and CAIC methods on both cancer data set allows us to correct for systematic bias while conserving or removing the copy number structure.

### Normalization of capture-Hi-C data with genomic duplication

**Fig. 6:**
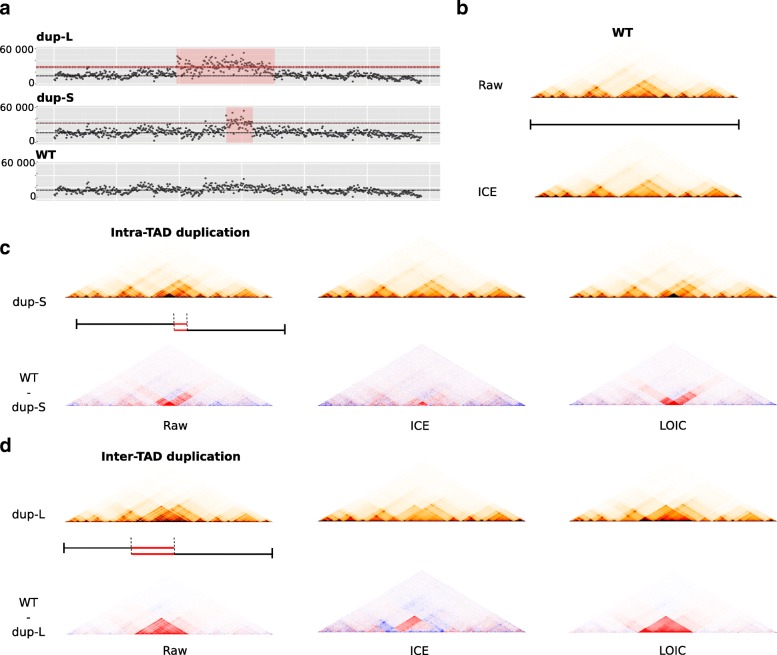
Duplication in capture-Hi-C and normalization. **a**. 1D profiles of capture-Hi-C wild-type sample (WT), with intra-TAD duplication (dup-S) or with inter-TAD (dup-L) duplication [10]. As expected, the duplication samples are characterized by twice more contacts at the duplicated sites. **b**. Raw and ICE normalized contact maps of WT sample. **c**. Normalization of the dup-S sample with the ICE and LOIC methods. The duplication effect is visualized by subtracting the normalized WT and dup-S contact maps. **d**. Same approach applied to the dup-L sample

In addition to cancer data, we also investigated the relevance of LOIC for the normalization of Hi-C data in samples with local structural events. Recently, Franke et al. [10] investigated the effect of local duplications on chromatin structure and, in particular, on the formation of new TADs. We processed the capture Hi-C data across the Sox9 locus and generated the raw and ICE contact maps at 10kb resolution. We focused our analysis on samples with inter-TAD (dup-S) and intra-TAD (dup-L) duplications. In the context of an inter-TAD duplication, Franke et al. [10] described the formation of a new domain in the duplicated region by comparing the raw contact maps of wild type (WT) and dup-L samples (Fig. 6c-d). Interestingly, when we compared the WT sample and the samples with the duplication events normalized by the ICE method, we observed that the ICE normalized maps do not allow duplication effects to be observed clearly. This is in agreement with our previous observations on cancer Hi-C data. We then applied our LOIC strategy following the observed 1D coverage profiles. As illustrated in Fig. 6, the LOIC normalization is able to remove systematic biases while keeping the copy number effect. The effects of both intra and inter-TAD duplication can therefore be clearly observed, validating the interest of our method for the study of local structural rearrangements.

### Removing the CNVs signal avoids misinterpretation of the chromosome compartment calling of cancer Hi-C data

We also explored the impact of CNVs and normalization on chromosome compartment calling. In intra-chromosomal contact maps, chromosome compartment profiles appear as checker-board-like interaction patterns, shifting from blocks with either high or low interaction frequency. Thus, chromosome compartments are usually detected using a Principal Component Analysis (PCA) on the correlation matrix of the distance-corrected intra-chromosomal contact maps. The first principal component then distinguishes the open (A) from closed (B) compartments [2] (see Additional file 1: Methods).

**Fig. 7:**
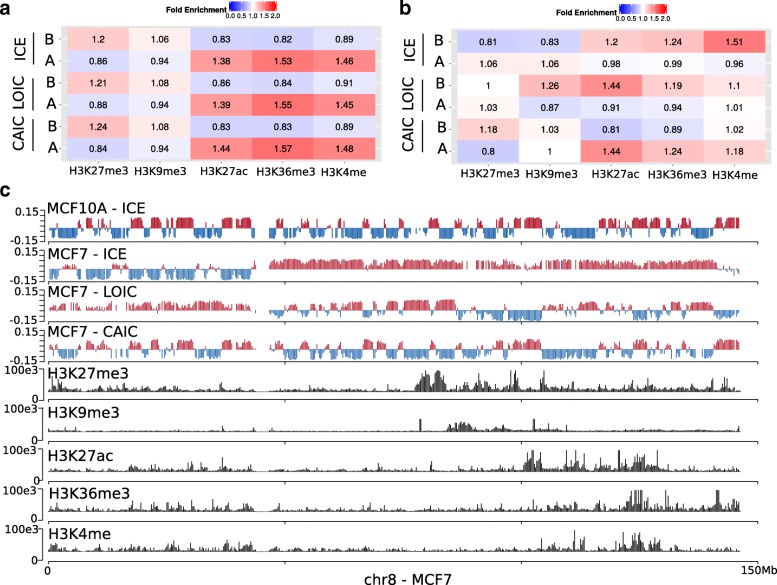
Detection of chromosome compartments. **a**. Genome-wide enrichment of ChIP-seq histone marks in open (A) or closed (B) compartments. Open compartments are enriched in open-chromatin marks, whereas closed compartments are enriched in repressive marks. The results are concordant genome-wide, whatever the normalization method applied. **b**. Histone marks enrichment on chromosome 8 of MCF7 sample. On this chromosome, the copy number has a strong impact on the compartment calling. **c**. Results of the compartment calling for the chromosome 8 of MCF7 sample (first principal component), together with normalized ChIP-seq tracks. Open chromatin domains are in red. Closed domains in blue

We performed compartment calling analysis on the MCF7 Hi-C data set normalized by ICE, LOIC, or CAIC methods and integrated the results with the histone marks data obtained from the ENCODE project [34]. Surprisingly, we observed that compartment calling is globally not affected by the CNVs on MCF7 data (Additional file 1: Figure S12) with around 8% of chromosome compartments switching from open to closed states (and vice-versa) according to the normalization method (Additional file 1: Figure S13a). We then assessed how the A/B compartments correlated with the active and repressive histone marks genome-wide (see Additional file 1: Methods). As expected, open compartments are associated with open-chromatin marks such as H3K27ac, H3K36me3 and H3K4me. Respectively, closed compartments are associated with repressive marks such as H3K27me3 or H3K9me3 (Fig. 7a).

Interestingly, looking at each chromosome independantly shows that, on the MCF7 data, the chromosome 8 harbours distinct compartment patterns according to the normalization method (Additional file 1: Figure S13a). In this case, it is clear that the copy number affects the PCA analysis and the compartment calling (Fig. 7b, c). We therefore conclude that it is important to correct for the copy number effect before running such analysis, and therefore that, by definition, the LOIC normalization method is not appropriate to this task. Applying the CAIC normalization method outperforms the other methods, resulting in a compartment profile which is well correlated with active/inactive histone marks, and which is closer to the normal MCF10A chromosome 8 profile (Fig.7b, c). The compartments pattern of the chromosome 8 extracted from the ICE normalized data is concordant with our previous conclusion that ICE is not appropriate to correct for CNVs, potentially leading to a wrong interpretation of the compartment profile. However, we also noticed that, in this case, the A/B compartments can be rescued by looking at the second principal component of the PCA.

Altogether, these results demonstrate that although compartment calling seems globally unaffected by copy number effects, applying the CAIC strategy to normalize the data improves the detection of A/B compartments, avoiding potential issues in their interpretation.

## Discussion

Chromosome conformation techniques provide a means to investigate links between the 3D organization of the genome and biological processes such as the functional and phenotypic effects of genomic variation in disease. Unsurprisingly, structural and copy number variations have also been observed to affect the genome architecture of cancer cells, perturbating TADs as well as the cell’s regulation [18, 35, 36], and as a result, the contact count maps of the genome. How do such genomic rearrangements impact on the performance of existing pipelines?

In order to better understand how large copy number variations can affect Hi-C data, we first propose a simple simulation model. From an existing diploid data set, our model is able to predict the effects of large copy number changes on interaction patterns by estimating a subsampling coefficient that can be applied in the presence of copy number variation. In addition to providing us with a theoretical framework to assess the enrichment and depletion of contact counts with respect to copy number variations, such a model can be used to assess how normalization methods and downstream analyses are affected by copy number variation. Our model is nevertheless simplistic and can be elaborated upon. For example, we consider that duplicated regions are non-tandemly rearranged events, which, in some cases, certainly underestimates the intra-chromosomal effect of copy number. In addition, the model does not integrate any biological knowledge and is therefore not designed to simulate changes due to the alteration of regulatory elements such as insulator regions. We also note that the subsampling strategy that we have applied here requires a high resolution diploid Hi-C data set as an input. However, as Hi-C sequencing depth increases, this should no longer be a limitation.

In our study, we go on to demonstrate that applying ICE (the most commonly used normalization method on Hi-C data) to data sets with abnormal copy number profiles leads to unbalanced corrections between amplified and lost regions and between inter and intra-chromosomal contacts. This unwanted effect can then lead to problematic results in downstream analysis (for example, in the identification of open and closed compartments). We therefore propose two new normalization methods that can remove systematic biases: LOIC, which preserves the effect of copy number variation, and CAIC, which removes the effects of copy number. In order to achieve this, both normalization methods require the identification of breakpoints. We thus also propose a segmentation procedure to directly extract the copy number signal and the location of breakpoints from the Hi-C contact maps. Although this step is crucial for the normalization and can be challenging for noisy samples, our procedure performs well on all the data set used in this study, including the normal diploid IMR90 sample. It also confirms that the Hi-C technique could become in the near-future a powerful approach to infer CNV profile in tumors.

Both CAIC and LOIC methods perform well on simulated and real Hi-C data sets, each fulfilling different needs. From a methodological point of view, we note that our assumption that the CNV effect is constant for each block delimited by a copy number change, cannot always be fulfilled, especially for very large genomic alterations. In such cases, modeling the CNV effect according to the distance between pairs of interacting loci could be an interesting extension to our current model.

The choice of which normalization method to use in Hi-C data analysis, will depend on the specific context and biological question. As an example, we demonstrate here that although chromosome compartment calling and PCA analysis are not dramatically affected by the copy number changes in MCF7 data, inappropriate normalization of the data can lead to incorrect interpretation of the results of such analysis. This effect can be even greater with other cell lines, as it is dependent on the copy number profile of the tumor (data not shown). This first experiment shows that CAIC, which removes the copy number biases, can enable existing analysis pipelines to be applied to cell lines with abnormal karyotypes. Yet, in other contexts, keeping the CNV informations could also be pertinant. For example, it may be of interest to examine the precise effect of local structural rearrangements and CNVs at smaller scale such as TADs level. As illustrated here with capture Hi-C data, applying our LOIC normalization procedure could be useful to remove systematic biases appropriately, while keeping the copy number structure.

## Conclusions

Taken together, the analyses covered here confirm that Hi-C can be a powerful technique to explore strutural variations on tumor samples and highlight the importance of using dedicated methods for the analysis of such data. The two new methods we introduce here (LOIC and CAIC) perform well on both simulated and real cancer Hi-C data sets, each answering different biological questions. As the application of Hi-C techniques to cancer and other samples to explore the 3D architecture of their genomes will continue to grow in the coming years, the importance of using the right techniques and developing more accurate tools to characterize such data sets appropiately is critical.

## Methods

Let us first introduce some notations. Given a segmentation of the genome into *n* genomic windows (or bins), Hi-C data can be summarized by a *n*-by-*n* symmetric matrix *C*, in which each row and column corresponds to a specific genomic loci and each entry *C*_*ij*_ the number of times loci *i* and *j* have been observed in contact. Let ${K \in \mathbb {R}^{n}}$ the copy number profile of the sample of interest, which we represent as a piecewise constant vector.

We denote by *s*(*i*,*j*) the genomic distance between the loci, defined as the number of base pairs between the center of the two loci; if *i* and *j* are not part of the same chromosome, we extend this definition by setting *s*(*i*,*j*)=*∞*. In this paper, we derive different ways to normalize the raw count matrix *C*: we denote by *N*^*y*^ the contact count matrix normalized with method *y* (e.g. *N*^ICE^ represents the ICE normalized count matrix).

### Simulation of cancer Hi-C data

Before we turn to how to appropriately model cancer Hi-C data, let us first review some terminology. In the literature, cis-contact counts refers to the contact counts between two loci of the same chromosome: this includes intra-chromosomal contact counts but also inter-chromosomal contact counts of homologous chromosomes. In this paper, we restrict the use of cis-contact counts to contact counts issued from the same DNA fragment, and we denote by “trans-homologous” (transH) interactions, the interactions between homologous chromosomes. Note that cis and transH contact counts are mostly indistinguishable (with the exception of allele-specific Hi-C) in Hi-C data hence the simplification of terminology usually used.

We now return to the problem at hand: how to simulate a contact count matrix *C*^sim^ of a cancer genome with abnormal copy number from a raw diploid contact count matrix *C*. In order to model the change in contact count abundances due to copy number variation, we first need to understand precisely which interactions are observed in the case of a simple diploid genome. For that purpose, we denote by *E*_*ij*_ the expected contact count between loci *i* and *j*, and $E^{\mathit {cis}}_{ij}$, $E^{\mathit {transH}}_{ij}$ and $E^{\mathit {trans}}_{ij}$ the expected cis, trans and transH-contact counts between *i* and *j*.

- if loci *i* and *j* belong to the same chromosome, the expected contact count *E*_*ij*_ is the sum of (1) cis-counts from either of the homologous chromosomes; (2) the transH-counts between the two homologous chromosomes:
  $$E_{ij} = 2 E^{\mathit{cis}}_{ij} + 2 E^{\mathit{transH}}_{ij} $$
- if loci *i* and *j* belong to different chromosomes, then the observed contact counts *E*_*ij*_ is the sum of either of the four possible *trans* interactions:
  $$E_{ij} = 4 E^{\mathit{trans}}_{ij}$$

This can be generalized to polyploid genome or to the context of chromosomal abnormalities (Additional file 1: Figure S1).

- if loci *i* and *j* belong to the same chromosome, let *k* be the number of cis interactions. If *i* and *j* belong to the same DNA segment, *k*=*K*_*i*_=*K*_*j*_. When *i* and *j* belong to different DNA segments, *k* could in theory take values between 0 and min(*K*_*i*_,*K*_*j*_). Here, we simulated the data with *k*=2, or *k*= min(*K*_*i*_,*K*_*j*_) if *K*_*i*_<2 or *K*_*j*_<2. Then
  $$E_{ij} = k E^{\mathit{cis}}_{ij} + (K_{i} K_{j} - k) E^{\mathit{transH}}_{ij} $$.
- if loci *i* and *j* belong to different chromosomes, then
  $$E_{ij} = K_{i}K_{j} E^{\mathit{trans}}_{ij} $$

Now that we have derived how contact counts are decomposed in terms of cis, transH and trans contact counts, we can leverage those relationships to simulate the effect of copy number variations on contact count matrices.

In order to derive a scaling factor *p*_*ij*_ which incorporates the copy number effect, we need to estimate $E^{\mathit {cis}}_{ij}$, $E^{\mathit {trans}}_{ij}$ and $E^{\mathit {transH}}_{ij}$, which is impossible without further assumptions. In practice, little is known about the probability of contact between homologous chromosomes, which is therefore difficult to estimate. However, we know that the chromosomes usually occupy their own space (chromosome territories) within the nucleus. We therefore make the assumption that all chromosomes are independent, and that the contact probability between homologous chromosomes can be estimated using the trans interaction between non-homologous chromosomes. We thus consider that *Eijtrans*=*EijtransH*=*E*^*trans*^.

From these relationships, we then calculate the scaling factor *p*_*ij*_ as following (recall that *E*_*ij*_ is the expected copy number between *i* and *j* on genome with abnormal chromosomal interactions):

- we estimate *E*^*trans*^ as the median trans-contact count;
- *Eijcis*=*C*_*ij*_−*E*^*trans*^;
- *E*_*ij*_ is estimated using the equations derived above;
- if loci *i* and *j* belong to the same chromosome, $p_{ij} = \frac {E_{ij}}{2E^{\mathit {cis}}_{ij} + 2E^{\mathit {trans}}}$
- if loci i and j belong to different chromosomes, $p_{ij} = \frac {K_{i} K_{j}}{4}$

We thus obtain, for each entry of the contact count matrix *C*_*ij*_, a ratio *p*_*ij*_ corresponding to the expected factor of enrichment or depletion of interactions for the loci *i* and *j*. In order to make the estimation of *p*_*ij*_ more robust, we estimate it constant per blocks of identical copy numbers by taking the median of the empirical values in each block. (See Additional file 1: Figure S11). Thus, the factor matrix *p* can be assumed to be block constant between regions of identical copy number variations. We thereby smooth *p* by computing the median scaling factor of block of similar copy number.

Finally, the simulated contact counts $C^{\text {sim}}_{ij}$ are generated by a binomial subsampling strategy of *C*_*ij*_ by a probability equal to $\frac {p_{ij}}{\text {max}(p_{ij})}$ [37]:

1 $$ C^{sim}_{ij} \sim B(C_{ij}, p_{ij})  $$

The reason for choosing a binomial subsampling as opposed to a simpler multiplication of the original Hi-C counts by a CNV-dependent factor, is that if *C*_*ij*_ follows a Poisson or Negative Binomial distribution, then $C^{sim}_{ij}$ follows the same distribution with modified expectation [37]. One limitation of this model is that the simulated counts can only be smaller than the original counts, which may be problematic if we start from small counts. It thus requires a diploid Hi-C data set with a sufficient sequencing depth to apply the downsampling strategy.

### Estimation of copy number from the contact count matrix

The copy number signal can be directly inferred from the Hi-C data in two steps. We first calculate the one-dimensional (1D) signal as the sum of genome-wide contact per bin, assuming that this signal reflects the true contact frequencies including the systematic Hi-C biases and the CNVs signal. We further calculate the GC content, the mappability and the effective fragment length of each bin end as already proposed [26]. The local genomic features of all chromosome bins are defined as the average of the corresponding features among all overlapping fragment ends. We then apply a Poisson regression model to correct the signal from GC content, mappability and fragment length, using the model proposed by Hu et al. [26]. The corrected profile is obtained by subtracting the fitted values to the observed data, and rescaled to be centered on 1. The normalized 1D data are then segmented using a pruned dynamic programming algorithm [38]. The segmented profile is smoothed with the GLAD package [39] in order to optimize the breakpoint locations and to remove false positives events. The segmentation is an important step of the method which may need to be adjusted according to the signal-to-noise ratio of the data. In this study, we apply the same parameters to all data sets and we consider the smoothed line after the segmentation as the Hi-C derived copy number profile.

To validate our estimation of copy number from Hi-C contact maps, we simulate an additional 100 data sets with varying copy number variations, as follows:

- draw a number of breakpoints along the genome;
- remove all breakpoints that fall into non mappable genome;
- draw the copy number coefficient from a poisson distribution of size 1. add 1 to these values;
- redraw any copy number coefficient that is equal to its neighbour;
- apply the simulation model presented above on these copy number profiles.

The copy number profiles are available as supplementary data.

### LOIC: Correcting technical biases of Hi-C cancer data

To normalize the contact count matrix, we adapt the ICE method proposed by Imakaev et al. [28] to incorporate the copy number effect. In particular, we use similar assumptions. First, the bias between two regions *i* and *j* can be decomposed as the product of two region-specific biases *β*_*ij*_=*β*_*i*_*β*_*j*_.

2 $$ \forall i,j\in[1,n]\,,\quad C_{ij} = \beta_{i} \beta_{j} N^{\text{LOIC}}_{ij} \,,  $$

where $\beta \in \mathbb {R}^{n}$ is a vector of bin-specific *biases*, such as gc-content, fragment lengths, mappability, etc.

Second, all copy-number identical regions interact as much:

3 $$  \forall i\in[\!1,n]\,,\quad \sum\limits_{j = 1}^{n} N^{LOIC}_{ij}= \frac{1}{|\{l\,|\, K_{l} = K_{i} \}|} \sum\limits_{l | K_{l} = K_{i}} \sum\limits_{j=1}^{n} C_{lj} \,.  $$

We refer to the second hypothesis as the “local equal-visibility assumption” to contrast it with ICE’s “equal-visibility assumption”: instead of enforcing an interaction profile constant across all the genome, we enforce an interaction profile constant for regions of identical copy number.

Similarly to Imakaev et al. [28], this problem can be solved exactly using matrix-balancing algorithms (under the assumption that the matrix is full decomposable [40]). Note that if there is no copy number variations, this boils down to solving exactly the same problem as ICE. On the other hand, in the presence of copy number variation, the resulting interaction profile will be a constant piecewise function, whose value depends on the copy number of the two loci.

In order to apply the proposed method, one needs to know a priori the set of bins with a given copy number or the copy number breakpoints. It can either be found via probing the samples to estimate it using specific technologies or through prior knowledge on the cell-line or sample studied. When none of these options are available, we can leverage the information provided by Hi-C data directly to estimate it.

### CAIC: Removing the copy number effect

The previous section describes how to normalize the raw contact counts matrix *C* to adjust for unwanted variations such as GC-content, mappability, fragment lengths, while keeping the copy number information. We now propose to estimate the effect of copy number variations on the contact count matrix to offer the possibility of removing it. We denote by *N*^*CAIC*^ the normalized contact count matrix where the CNV effect has been removed.

We assume that the copy number effect for each pair of loci is identical for element with identical copy-number variations. This reflects that the copy-number effect between loci *i* and *j* is related to the amount of genetic material of those two regions, and thus identical between all pairs with similar copy-number variations. We can thus model the normalized contact count matrix as the product of a block-constant matrix *B* and corrected matrix *N*^CAIC^: $N^{CAIC}_{ij} = B_{ij} N^{\text {LOIC}}_{ij}$, where each block is a function of the copy number in *i* and in *j*.

In addition, we assume that, on average, each pair of loci interacts roughly the same way as any pair of loci at the same genomic distance *s*:

4 $$ N^{CAIC}_{l,m} \simeq \mathit{e}(s(l, m))\,,  $$

where *e*(*s*) is the expected contact count at genomic distance *s*. We leverage this assumption to cast an optimization problem:

$$\begin{array}{ccll} \underset{\mathit{e}, B}{\text{min}} & \underset{i, j}\sum \left(N^{LOIC}_{ij} - B_{ij} \mathit{e}(s(i, j))\right)^{2} \\ \text{subject to} & B \quad \text{is block-constant} \\ & \mathit{e} \quad \text{decreasing}\\ \end{array} $$

We solve this optimization problem genome-wide by iteratively estimating the block constant matrix *B* and the expected counts function *e* using an isotonic regression. Note that the trans estimation can be done jointly on the whole genome independently from the cis estimation.

## Additional file

Additional file 1 Supplementary Methods and Figures. This file contains supplemental methods, **Tables S1-S3**, and **Figures S1-S13** (PDF 19,614 kb)

## Abbreviations

1D: One dimension
CNVs: Copy number variants
CAIC: CNV-adjusted iterative correction
ICE: Iterative correction and eigenvector decomposition
LOIC: LOcal iterative correction
PCA: Principal component analysis
TADs: Topological associated domains

## References

[1] Bonev B, Cavalli G. Organization and function of the 3D genome. Nat Rev Genet. 2016; 17:661–78. 10.1038/nrg.2016.112. 10.1038/nrg.2016.112 27739532

[2] Lieberman-Aiden E, van Berkum NL, Williams L, Imakaev M, Ragoczy T, Telling A, Amit I, Lajoie BR, Sabo PJ, Dorschner MO, Sandstrom R, Bernstein B, Bender MA, Groudine M, Gnirke A, Stamatoyannopoulos J, Mirny LA, Lander ES, Dekker J. Comprehensive mapping of long-range interactions reveals folding principles of the human genome. Sci (NY). 2009; 326:289–93. 10.1126/science.1181369. 10.1126/science.1181369 PMC2858594 19815776

[3] Rao SSP, Huntley MH, Durand N, Neva C, Stamenova EK, Bochkov ID, Robinson JT, Sanborn AL, Machol I, Omer AD, Lander ES, Aiden EL (2014). A 3D map of the human genome at kilobase resolution reveals principles of chromatin looping. Cell.

[4] Dixon JR, Jung I, Selvarajv S, Shen Y, Antosiewicz-Bourget JE, Lee AY, Ye Z, Kim A, Rajagopal N, Xie W, Diao Y, Liang J, Zhao H, Lobanenkov VV, Ecker JR, Thomson JA, Ren B. Chromatin architecture reorganization during stem cell differentiation. Nature. 2015; 518:331–6. 10.1038/nature14222. 10.1038/nature14222 PMC4515363 25693564

[5] Barutcu AR, Lajoie BR, McCord RP, Tye CE, Hong D, Messier TL, Browne G, van Wijnen AJ, Lian JB, Stein JL, Dekker J, Imbalzano AN, Stein GS. Chromatin interaction analysis reveals changes in small chromosome and telomere clustering between epithelial and breast cancer cells. Genome Biol. 2015; 16:214. 10.1186/s13059-015-0768-0. 10.1186/s13059-015-0768-0 PMC4587679 26415882

[6] Nora EP, Lajoie BR, Schulz EG, Giorgetti L, Okamoto I, Servant N, Piolot T, van Berkum NL, Meisig J, Sedat J, Gribnau J, Barillot E, Blüthgen N, Dekker J, Heard E. Spatial partitioning of the regulatory landscape of the X-inactivation centre. Nature. 2012; 485:381–5. 10.1038/nature11049. 10.1038/nature11049 PMC3555144 22495304

[7] Dixon JR, Selvaraj S, Yue F, Kim A, Li Y, Shen Y, Hu M, Liu JS, Ren B. Topological domains in mammalian genomes identified by analysis of chromatin interactions. Nature. 2012; 485:376–80. 10.1038/nature11082. 10.1038/nature11082 PMC3356448 22495300

[8] Bouwman BAM, de Laat W. Getting the genome in shape: the formation of loops, domains and compartments. Genome Biol. 2015; 16:154. 10.1186/s13059-015-0730-1. 10.1186/s13059-015-0730-1 PMC4536798 26257189

[9] Krijger PHL, de Laat W. Regulation of disease-associated gene expression in the 3D genome. Nat Rev Mol Cell Biol. 2016; 17:771–82. 10.1038/nrm.2016.138. 10.1038/nrm.2016.138 27826147

[10] Franke M, Ibrahim DM, Andrey G, Schwarzer W, Heinrich V, Schöpflin R, Kraft K, Kempfer R, Jerković I, Chan W-L, Spielmann M, Timmermann B, Wittler L, Kurth I, Cambiaso P, Zuffardi O, Houge G, Lambie L, Brancati F, Pombo A, Vingron M, Spitz F, Mundlos S. Formation of new chromatin domains determines pathogenicity of genomic duplications. Nature. 2016; 538:265–9. 10.1038/nature19800. 10.1038/nature19800 27706140

[11] Lupiáñez DG, Spielmann M, Mundlos S. Breaking TADs: How Alterations of Chromatin Domains Result in Disease. Trends in genetics : TIG. 2016; 32:225–37. 10.1016/j.tig.2016.01.003. 10.1016/j.tig.2016.01.003 26862051

[12] Ciriello G, Miller ML, Aksoy BA, Senbabaoglu Y, Schultz N, Sander C. Emerging landscape of oncogenic signatures across human cancers. Nat Genet. 2013; 45:1127–13. 10.1038/ng.2762. 10.1038/ng.2762 PMC4320046 24071851

[13] Vogelstein B, Papadopoulos N, Velculescu VE, Zhou S, Diaz LA, Kinzler KW. Cancer genome landscapes. Sci (NY). 2013; 339:1546–58. 10.1126/science.1235122. 10.1126/science.1235122 PMC3749880 23539594

[14] Plass C, Pfister SM, Lindroth AM, Bogatyrova O, Claus R, Lichter P. Mutations in regulators of the epigenome and their connections to global chromatin patterns in cancer. Nat Rev Genet. 2013; 14:765–80. 10.1038/nrg3554. 10.1038/nrg3554 24105274

[15] Taberlay PC, Statham AL, Kelly TK, Clark SJ, Jones PA. Reconfiguration of nucleosome-depleted regions at distal regulatory elements accompanies DNA methylation of enhancers and insulators in cancer. Genome Res. 2014; 24:1421–32. 10.1101/gr.163485.113. 10.1101/gr.163485.113 PMC4158760 24916973

[16] Losada A. Cohesin in cancer: chromosome segregation and beyond. Nat Rev Cancer. 2014; 14:389–93. 10.1038/nrc3743. 10.1038/nrc3743 24854081

[17] Gröschel S, Sanders MA, Hoogenboezem R, de Wit E, Bouwman BAM, Erpelinck C, van der Velden VHJ, Havermans M, Avellino R, van Lom K, Rombouts EJ, van Duin M, Döhner K, Beverloo HB, Bradner JE, Döhner H, Löwenberg B, Valk PJM, Bindels EMJ, de Laat W, Delwel R. A single oncogenic enhancer rearrangement causes concomitant EVI1 and GATA2 deregulation in leukemia. Cell. 2014; 157:369–81. 10.1016/j.cell.2014.02.019. 10.1016/j.cell.2014.02.019 24703711

[18] Taberlay PC, Achinger-Kawecka J, Lun ATL, Buske FA, Sabir K, Gould CM, Zotenko E, Bert SA, Giles KA, Bauer DC, Smyth GK, Stirzaker C, O’Donoghue SI, Clark SJ. Three-dimensional disorganization of the cancer genome occurs coincident with long-range genetic and epigenetic alterations. Genome Res. 2016; 26:719–31. 10.1101/gr.201517.115. 10.1101/gr.201517.115 PMC4889976 27053337

[19] Hnisz D, Weintraub AS, Day DS, Valton A-L, Bak RO, Li CH, Goldmann J, Lajoie BR, Fan ZP, Sigova AA, Reddy J, Borges-Rivera D, Lee TI, Jaenisch R, Porteus MH, Dekker J, Young RA. Activation of proto-oncogenes by disruption of chromosome neighborhoods. Science (NY). 2016; 351:1454–8. 10.1126/science.aad9024. 10.1126/science.aad9024 PMC4884612 26940867

[20] Weischenfeldt J, Dubash T, Drainas AP, Mardin BR, Chen Y, Stütz AM, Waszak SM, Bosco G, Halvorsen AR, Raeder B, Efthymiopoulos T, Erkek S, Siegl C, Brenner H, Brustugun OT, Dieter SM, Northcott PA, Petersen I, Pfister SM, Schneider M, Solberg SK, Thunissen E, Weichert W, Zichner T, Thomas R, Peifer M, Helland A, Ball CR, Jechlinger M, Sotillo R, Glimm H, Korbel JO. Pan-cancer analysis of somatic copy-number alterations implicates IRS4 and IGF2 in enhancer hijacking. Nat Genet. 2017; 49:65–74. 10.1038/ng.3722. 10.1038/ng.3722 PMC5791882 27869826

[21] Beroukhim R, Zhang X, Meyerson M. Copy number alterations unmasked as enhancer hijackers. Nat Genet. 2016; 49:5–6. 10.1038/ng.3754. 10.1038/ng.3754 28029156

[22] Flavahan WA, Drier Y, Liau BB, Gillespie SM, Venteicher AS, Stemmer-Rachamimov AO, Suvà ML, Bernstein BE. Insulator dysfunction and oncogene activation in IDH mutant gliomas. Nature. 2016; 529:110–4. 10.1038/nature16490. 10.1038/nature16490 PMC4831574 26700815

[23] Ramani V, Shendure J, Duan Z. Understanding spatial genome organization: Methods and insights. Genomics, Proteomics Bioinforma. 2016; 14:7–20. 10.1016/j.gpb.2016.01.002. 10.1016/j.gpb.2016.01.002 PMC4792841 26876719

[24] Yaffe E, Tanay A. Probabilistic modeling of Hi-C contact maps eliminates systematic biases to characterize global chromosomal architecture. Nat Genet. 2011; 43:1059–65. 10.1038/ng.947. 10.1038/ng.947 22001755

[25] Ay F, Noble WS. Analysis methods for studying the 3D architecture of the genome. Genome Biol. 2015; 16:183. 10.1186/s13059-015-0745-7. 10.1186/s13059-015-0745-7 PMC4556012 26328929

[26] Hu M, Deng K, Selvaraj S, Qin Z, Ren B, Liu JS (2012). HiCNorm: removing biases in Hi-C data via Poisson regression. Bioinformatics.

[27] Cournac A, Marie-Nelly H, Marbouty M, Koszul R, Mozziconacci J. Normalization of a chromosomal contact map. BMC Genomics. 2012; 13:436. 10.1186/1471-2164-13-436. 10.1186/1471-2164-13-436 PMC3534615 22935139

[28] Imakaev M, Fudenberg G, McCord RP, Naumova N, Goloborodko A, Lajoie BR, Dekker J, Mirny LA (2012). Iterative correction of Hi-C data reveals hallmarks of chromosome organization. Nat Methods.

[29] Wu H-J, Michor F. A computational strategy to adjust for copy number in tumor Hi-C data. Bioinformatics (Oxford, England). 2016; 32:3695–701. 10.1093/bioinformatics/btw540. 10.1093/bioinformatics/btw540 PMC6078171 27531101

[30] Harewood L, Kishore K, Eldridge MD, Wingett S, Pearson D, Schoenfelder S, Collins VP, Fraser P. Hi-c as a tool for precise detection and characterisation of chromosomal rearrangements and copy number variation in human tumours. Genome Biol. 2017; 18:125. 10.1186/s13059-017-1253-8. 10.1186/s13059-017-1253-8 PMC5488307 28655341

[31] Boeva V, Popova T, Bleakley K, Chiche P, Cappo J, Schleiermacher G, Janoueix-Lerosey I, Delattre O, Barillot E (2012). Control-freec: a tool for assessing copy number and allelic content using next-generation sequencing data. Bioinformatics.

[32] Le Dily F, Bau D, Pohl A, Vicent GP, Serra F, Soronellas D, Castellano G, Wright RHG, Ballare C, Filion G, Marti-Renom MA, Beato M. Distinct structural transitions of chromatin topological domains correlate with coordinated hormone-induced gene regulation. Genes Dev. 2014; 28:2151–62. 10.1101/gad.241422.114. 10.1101/gad.241422.114 PMC4180976 25274727

[33] Servant N, Varoquaux N, Lajoie BR, Viara E, Chen CJ, Vert JP, Heard E, Dekker J, Barillot E (2015). HiC-Pro: an optimized and flexible pipeline for Hi-C data processing. Genome Biol.

[34] Consortium. TEP (2012). An integrated encyclopedia of DNA elements in the human genome. Nature.

[35] Valton A-L, Dekker J. Tad disruption as oncogenic driver. Curr Opin Genet Dev. 2016; 36:34–40. 10.1016/j.gde.2016.03.008. 10.1016/j.gde.2016.03.008 PMC4880504 27111891

[36] Wu P, Li T, Li R, Jia L, Zhu P, Liu Y, Chen Q, Tang D, Yu Y, Li C (2017). 3d genome of multiple myeloma reveals spatial genome disorganization associated with copy number variations. Nat Commun.

[37] Wiuf C, Stumpf PH. Binomial subsampling. Proc. R. Soc. A. 2006; 462:1181–95. 10.1098/rspa.2005.1622.

[38] Picard F, Lebarbier E, Hoebeke M, Rigaill G, Thiam B, Robin S. Joint segmentation, calling, and normalization of multiple CGH profiles. Biostatistics. 2011; 12(3):413–28. https://doi.org/:10.1093/biostatistics/kxq076. 10.1093/biostatistics/kxq076 21209153

[39] Hupé P, Stransky N, Thiery J, Radvanyl F, Barillot E. GLAD: Gain and Loss Analysis of DNA. Bioinformatics. 2004; 20(18):3413–22. 10.1093/bioinformatics/bth418. 10.1093/bioinformatics/bth418 15381628

[40] Sinkhorn R, Knopp P (1967). Concerning nonnegative matrices and doubly stochastic matrices. Pacific J Math.

