# Supplementary material for: Effective normalization for copy number variation in Hi-C data
Source: BMC Bioinformatics. 2018 Sep 6;19:313. doi: 10.1186/s12859-018-2256-5 (PMC6127909; doi:10.1186/s12859-018-2256-5)
Supplement: Supplementary file 1 — Supplementary Methods and Figures. This file contains supplemental methods, Tables S1-S3, and Figures S1-S13 (PDF 19,614 kb) [file 12859_2018_2256_MOESM1_ESM.pdf]

Effective normalization for copy number variation in Hi-C data

N. Servant [1,2,3,*,†], N. Varoquaux [4,5,†], E. Heard [6], E. Barillot [1,2,3], JP. Vert [3,1,2,6]

June 4, 2018

[1]Institut Curie, PSL Research University, F-75005, Paris, France, [2]INSERM, U900, F-75005, Paris, France, [3]Mines ParisTech, PSL Research University, CBIO-Centre for Computational Biology, F-75006, Paris, France, [4]Department of Statistics, University of California, Berkeley, USA, [5]Berkeley Institute for Data Science, Berkeley, USA, [6]Institut Curie, PSL Research University, CNRS UMR3215, INSERM U934, F-75005, Paris, France, [7]Ecole Normale Supérieure, PSL Research University, Department of Mathematics and Applications, F-75005, Paris, France.

1 Supplementary methods

1.1 Data

We evaluate the copy number effect on Hi-C data using publicly available dataset (see Table S1).
Simulation data are generated using the IMR90_CCL6 data from **?** (GSE63525). Real data from T47D and MCF7 breast cancer cell lines, as well capture Hi-C data, are used to further validate our results.

Datatype	Sample	ID	Reference	Description
Hi-C	IRM90_CCL6	GSE63525	[?]	Diploid IMR90 Hi-C data at high resolution
Hi-C	Simulation 1	-	-	Highly rearranged simulated data derived from IMR90 Hi-C data
Hi-C	Simulation 2	-	-	Aneuploidy simulated data derived from IMR90 Hi-C data
Hi-C	T47D	GSE53463	[?]	T47D breast cancer Hi-C data
Hi-C	MCF7	GSE66733	[?]	MCF7 breast cancer Hi-C data
Hi-C	MCF10-A	GSE6673	[?]	MCF10-A nearly-diploid Hi-C data
Capture Hi-C	Mouse E12.5 limb buds	GSE78072	[?]	Capture Hi-C at the Sox9/Kcnj locus
ChIP-seq	MCF7 H3K27me3	ENCODE	-	H3K27me3 signal track
ChIP-seq	MCF7 H3K09me3	ENCODE	-	H3K09me3 signal track
ChIP-seq	MCF7 H3K27ac	ENCODE	-	H3K27ac signal track
ChIP-seq	MCF7 H3K36me3	ENCODE	-	H3K36me3 signal track
ChIP-seq	MCF7 H3K04me	ENCODE	-	H3K27me3 signal track

Table S1: Description of data used in this study.

*To whom correspondence should be addressed
†Equally Contributed

1.2 Data Processing

All raw Hi-C data are processed with the HiC-Pro pipeline v2.8.0 [?] up to normalized ICE contact maps. Iterative corrections are processed using the iced python package (v0.4.2), after removing the 2% low coverage rows and columns.
Intra-chromosomal contact maps are annotated as previously described, by summarizing for each bin the GC content, effective fragment lengths and mappability [?]. In order to explore the bias in cis contact maps, we first split each feature into 20 bins of equal size. The cis contact maps are then normalized by the expected counts based on genomic distance, in order to generate the observed/expected (O/E) maps (See Section 1.3. For each feature bin, we then calculate the mean contact frequency over all cis-O/E maps.

Affymetrix SNP6.0 analysis raw data are normalized by technology specific software to extract signal for each probe. The obtained copy number profile is smoothed by a segmentation algorithm to remove noise and detect breakpoints. A similar process is applied on the allelic frequency probe [?]. The combined type of probes allow getting an estimate of absolute copy number for each probe, sample cellularity and tumour ploidy.

1.3 Computing Observed/Expected (O/E) matrices

We estimate the "Expected" matrices as follow. First, for each genomic distance s, compute the mean contact count interaction. then, apply an isotonic regression to enforce that the expected count decease as a function of the genomic distance. We set the trans expected count to the average trans contact count. We thus obtain the function $e(s)$, described in the Methods. We can then compute the O/E matrices:

$$O/E_{ij} = \frac{C_{ij}}{e(s(i,j))} \tag{1}$$

1.4 Estimating the error

To assess the ability of different methods to normalize abnormal karyotype data, we need quantitative measures of similarities between contact maps. We propose to use three measures, with different properties. First, we compute the O/E matrices as described before: this normalizes both for different coverage and the structure induced by structural properties of the DNA. We then compute the "block-average" of the matrix: between each copy-number breakpoint, we compute the mean of the matrix. We denote by $\bar{C}$ the block-average of C. We then compare this resulting matrix with the "block-average" of the ground-truth G in three different manners: the ℓ_1 error, ℓ_2 error and $\ell_{\max}$.

$$\ell_1(C) = \sum_{i,j}(|\bar{C}_{ij} - \bar{G}_{ij}|)$$
$$\ell_2(C) = \sum_{i,j}(|\bar{C}_{ij} - \bar{G}_{ij}|^2)$$
$$\ell_{\max}(C) = \max_{i,j}(|\bar{C}_{ij} - \bar{G}_{ij}|)$$

In addition, we refer to the difference between block-average matrix of the ground truth and the normalize count as the "block average error matrix".

1.5 Chromosome compartments calling

Chromosome compartments calling is performed as previously described on 250Kb contact maps [?] using the HiTC R package [?] and the *pca.hic* function. First, O/E maps are estimated, thus normalizing contact counts for the structural effect of the genomic distance. Then, Principal Component Analysis (PCA) on the pearson correlation of the O/E matrices is applied. Active and inactive compartments are respectively assigned based on genome-wide gene density.

We further assess the enrichment of chromatin marks within A/B chromosomal compartments. To do so, the ChIP-seq signal is binned into 100 kb bins and normalized by the copy number signal. We then calculate the enrichment fold as the median of the signal track in bins within the cluster of interest, divided by the median of the signal track across all bins. The fold enrichment is calculated genome-wide, or for each chromosome independantly.

1.6 Capture-C analysis

The raw sequencing data were downloaded from GEO (GSE78072) and processed using HiC-Pro (v2.8.0). In order to compare the different contact maps, we applied the strategy proposed by Franke et al. For each map, we excluded

the regions involved in the duplications and calculated a scaling factor for each map (sum of contacts/10^6). Each contact matrix was then scaled by its scaling factor before subtraction.

2 Supplementary tables

	MCF7	MCF7 simulated	MCF10A
MCF7	1.000		
MCF7 simulated	0.877	1.000	
MCF10A	0.596	0.662	1.000

Table S2: **Correlation of 1D profiles.**
Spearman correlation of raw 1D profiles of MCF7, MFC7 simulated and MCF10A data. The correlation of MCF7 simulated data with MCF7 real data is higher than other baselines based on MCF7 and MCF10A correlations.

	ICE			CAIC		
	ℓ_1	ℓ_2	$\ell_{\max}$	ℓ_1	ℓ_2	$\ell_{\max}$
Aneuploid data set	2.321×10^6	4.574×10^5	0.313	2.291×10^6	3.532×10^5	0.185
Highly rearranged data set	3.984×10^6	8.832×10^5	1.344	2.055×10^6	2.536×10^5	0.360

Table S3: **Errors on the simulated data set.**

3 Supplementary figures

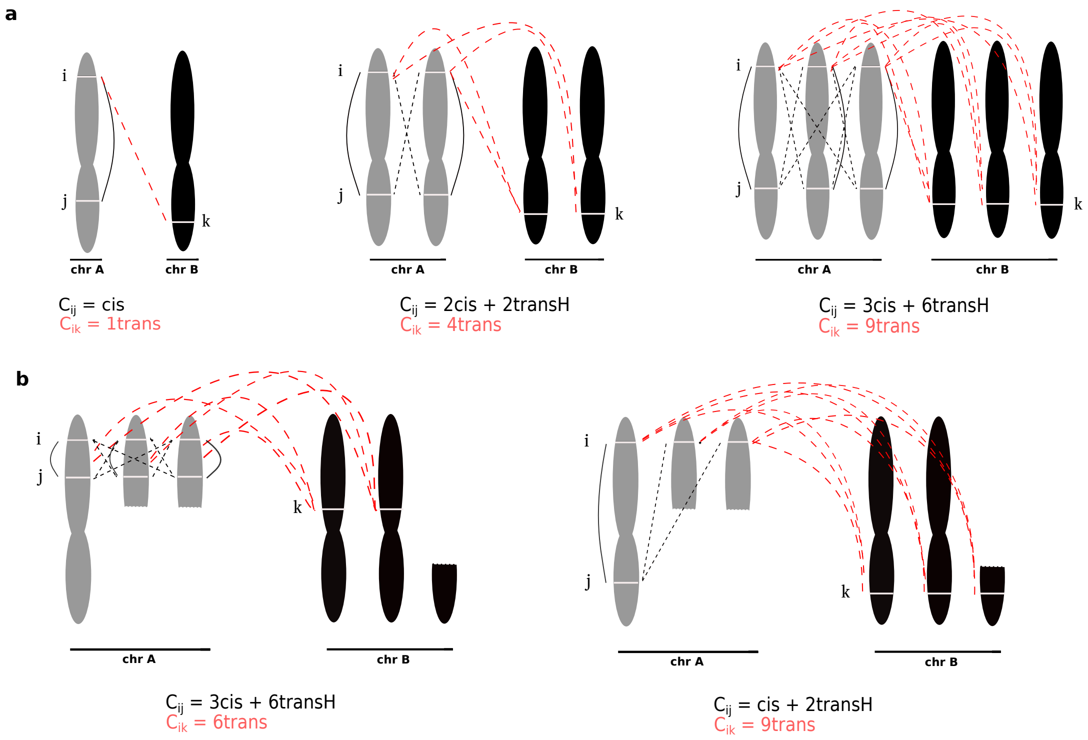

Figure S1: **Case study of simulated cancer Hi-C data.**
a. Example of contact frequency decomposition in the context of polyploid genome. **b.** Extension to local copy number rearrangement, when the two loci are on the same DNA segment (left) and on different segments (right).

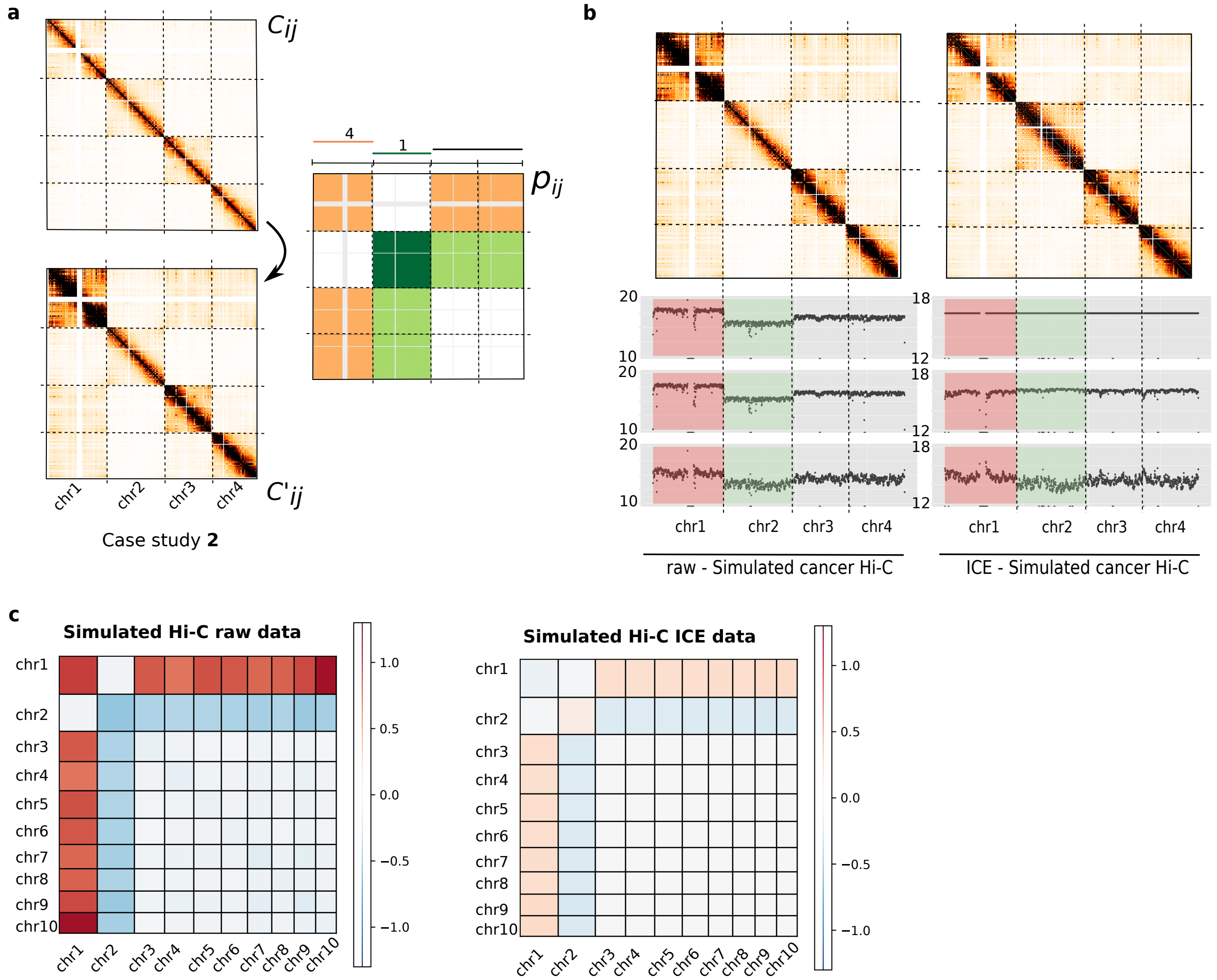

Figure S2: **Simulation of aneuploidy data.**

a. We explore different simulation scenarios based on different copy number profiles. This simulation is only based on gain or loss of complete chromosomes, therefore modeling aneuploidy effect. **b.** Applying the iterative correction on aneuploidy data should efficiently correct intra-chromosomal data from biases. However, the method does not properly rescale the inter-chromosomal contacts of different chromosomes. **b.** Block-average error matrix of simulated raw and ICE cancer data. As previously observed, the iterative correction does not allow to correct for segmental copy number bias.

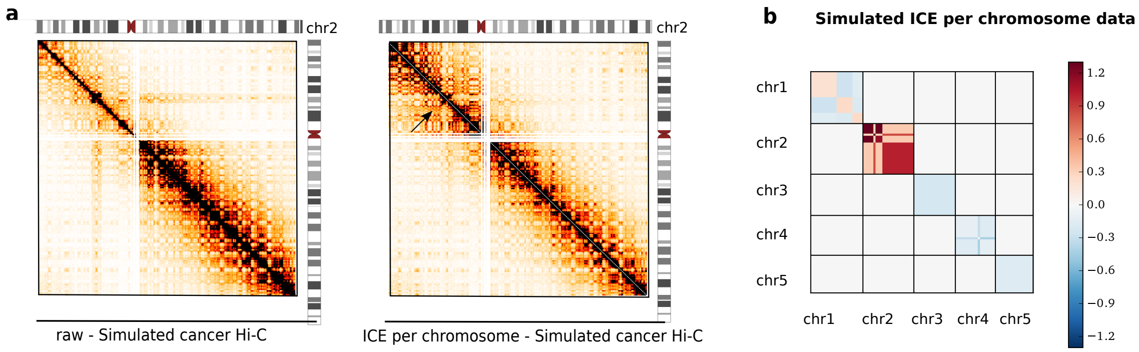

Figure S3: **ICE normalization per chromosome.**
a. Example of chromosome 2 contact maps before and after iterative correction per chromosome. **b.** Applying the iterative correction per chromosome on the intra-chromosomal data gives better results than the genome-wide approach. However, we can see that some biases still remains locally in the short-range contacts.

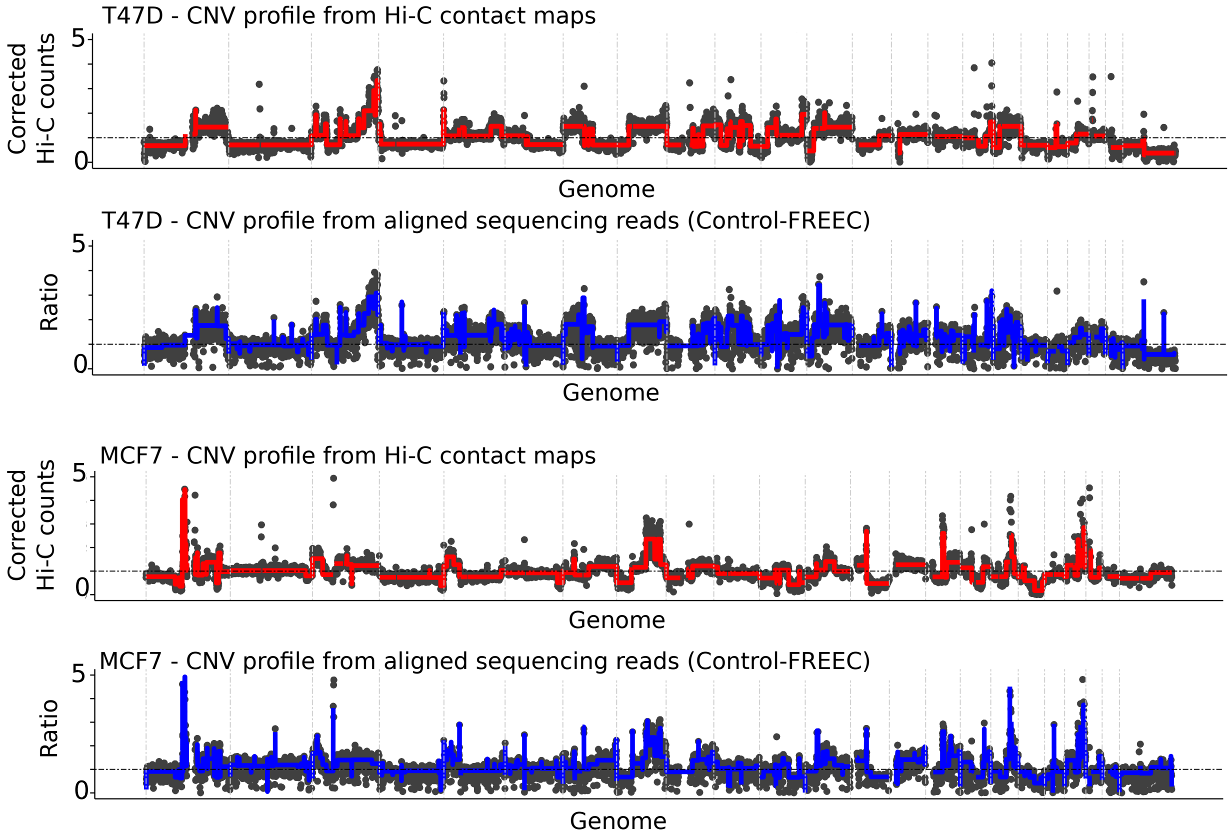

Figure S4: **CNV profile estimated from Hi-C contact maps or genome-wide reads mapping** Comparison of our method for CNV estimation from Hi-C contact maps, with method based on reads mapping (Control-FREEC, default parameters). We observed a good correlation between both methods (MCF7 spearman cor=0.888, T47D spearman cor=0.855) therefore validating our approach.

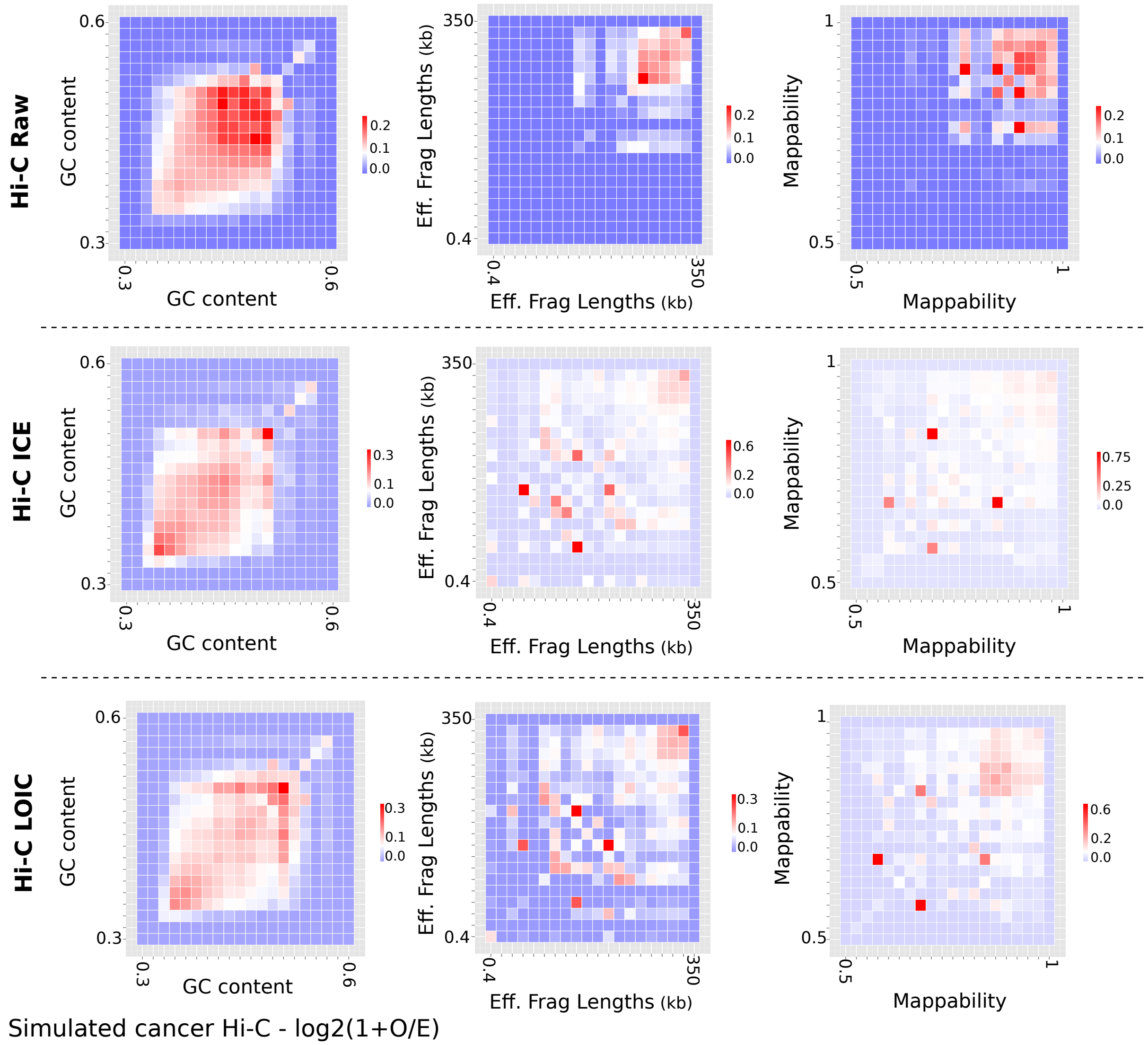

Figure S5: **Systematic biases in Hi-C cis experiment.**
The GC content, effective fragment length and mappability features were calculated for each 500 Kb bin. Each annotation feature was then splitted into 20 bins of equal size. The cis contact maps of raw, ICE and LOIC data were then normalized by the expected counts based on genomic distance, in order to generate the observed/expected (O/E) maps. For each feature bin, we then represented as a heatmap the mean contact frequency (log2).

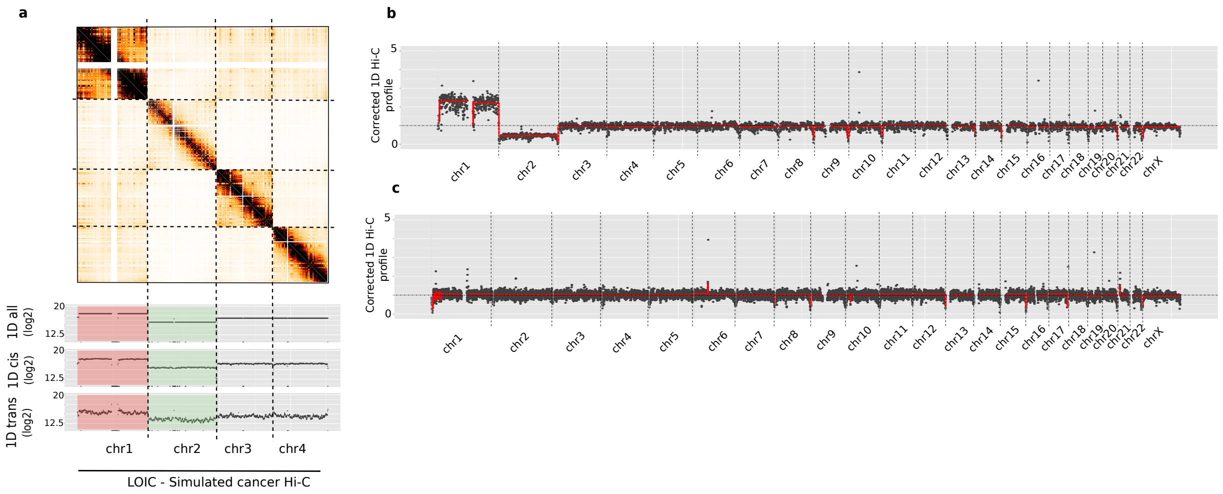

Figure S6: **Application of LOIC on simulated aneuploidy and diploid Hi-C data.**
a. 1D profile of Hi-C data after LOIC normalization. As expected, the LOIC strategy allows to conserve the copy number information both in cis and trans contacts. **b.** Segmentation results of simulated aneuploid Hi-C data. **c.** Segmentation profile of diploid IMR90 Hi-C data. For diploid data, the segmentation profile is mainly flat along the genome. LOIC procedure should therefore gives very similar results as compared to the ICE method.

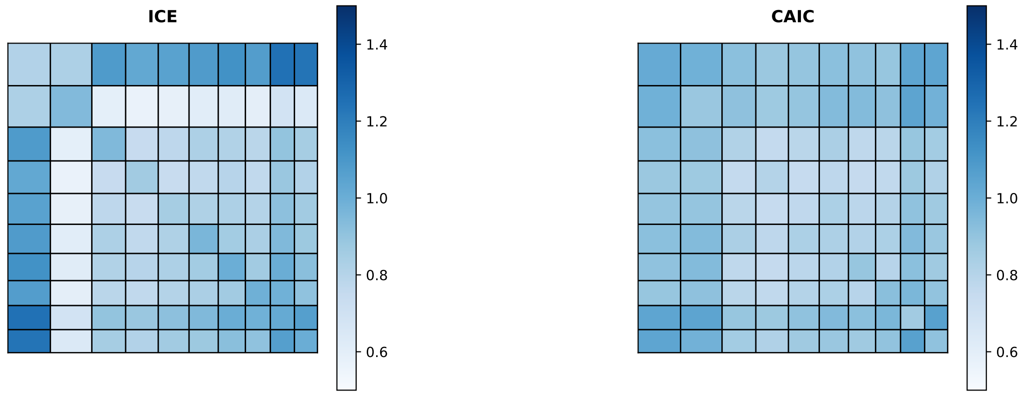

Figure S7: **“block-average” matrices for aneuploid simulated data set.**
We observe that ICE introduces a bias when correcting aneuploid data, mostly by over-correcting depleted trans regions. CAIC yields as expected a nearly uniform matrix.

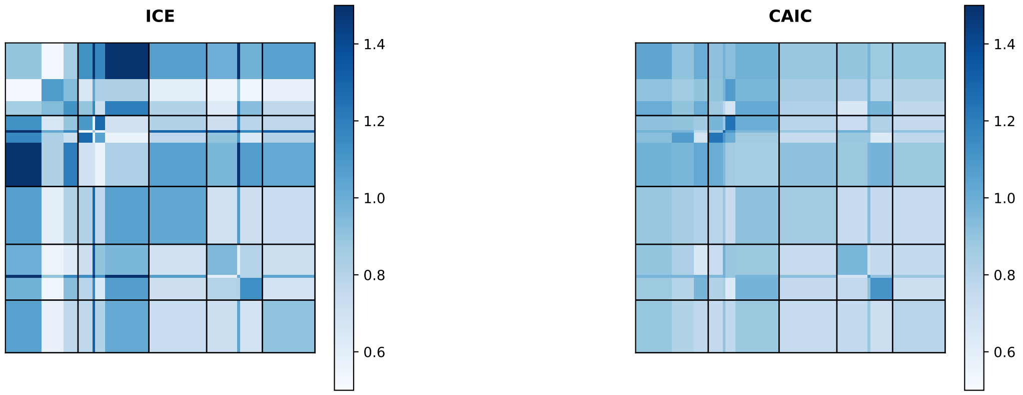

Figure S8: **"block-average" matrices for highly rearranged simulated data set.** We observe that ICE introduces a bias when correcting abnormal karyotype data.

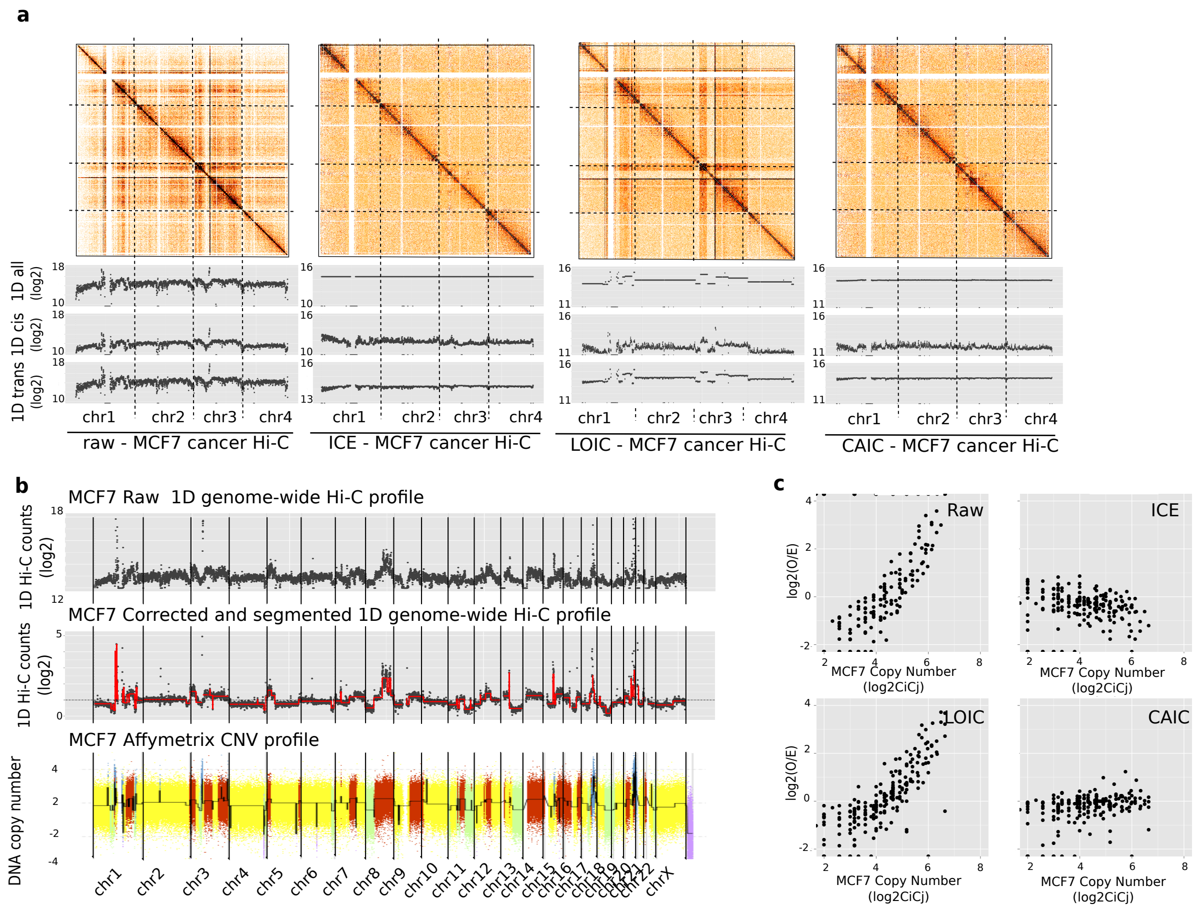

Figure S9: **Normalization of MCF7 Hi-C data. a.** Hi-C contact maps (250Kb resolution) of the first four chromosomes of MCF7 cancer Hi-C sample. As already shown on simulated and T47D data, we observed that ICE introduces a bias on normalized data. We then applied the LOIC and CAIC normalization approches to correct for systematic biases while removing or keeping the CNVs effect. **b.** In order to estimate the copy number signal from the Hi-C data, we applied a correction and segmentation method to the 1D profile. The inferred copy number signal from the Hi-C data are highly correlated with the copy number profile from Affymetrix SNP6.0 array. **c.** Relationship between contact frequencies with copy number on raw and normalized Hi-C data.

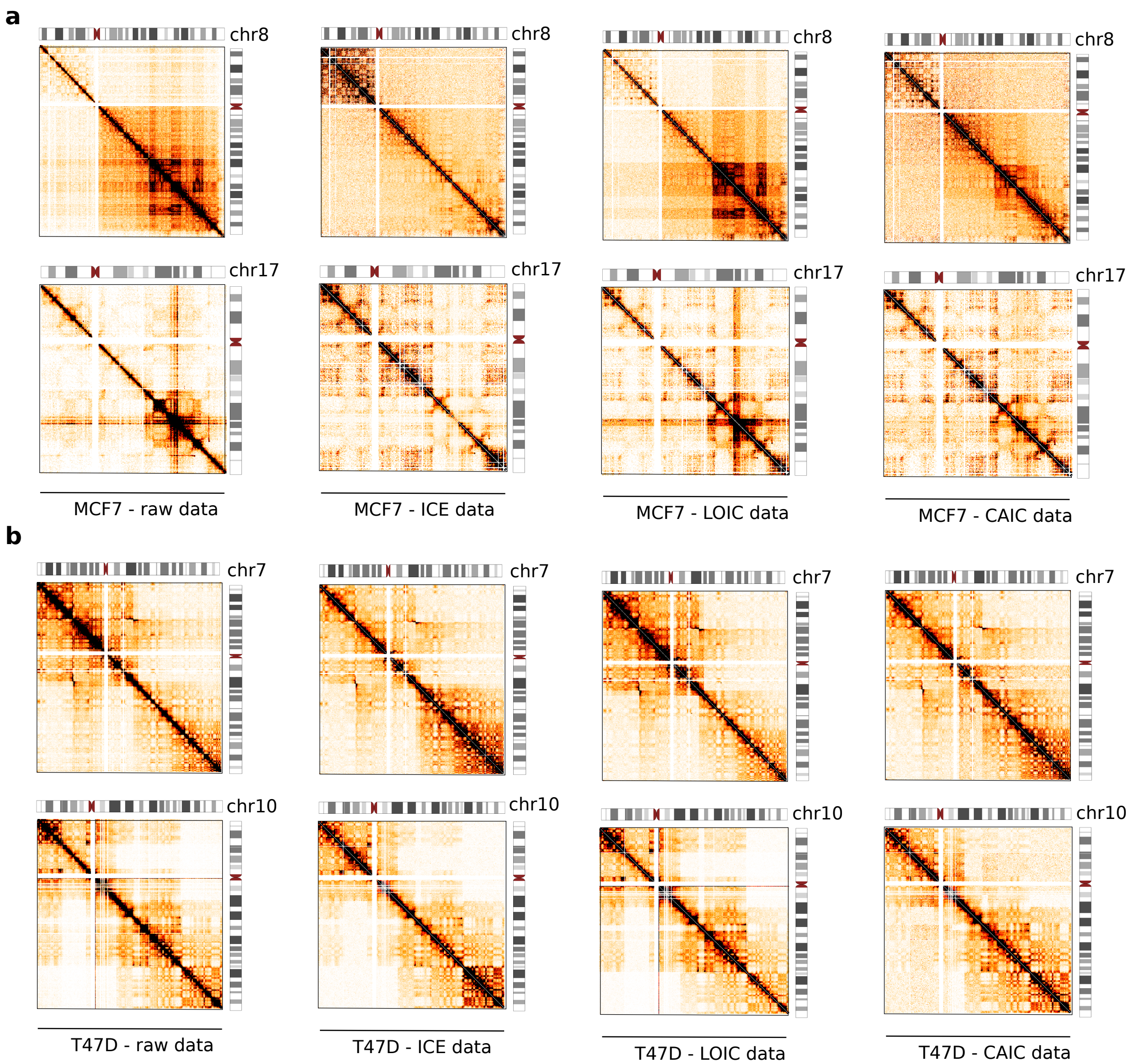

Figure S10: **Normalized intra-chromosomal contact maps.** **a.** Intra-chromosomal contact maps of chromosome 8 and 17 from MCF7 data, not normalized or normalized using the ICE, LOIC or CAIC method. **b.** Intra-chromosomal contact maps of chromosome 10 and 7 from T47D data, not normalized or normalized using ICE, LOIC or CAIC method.

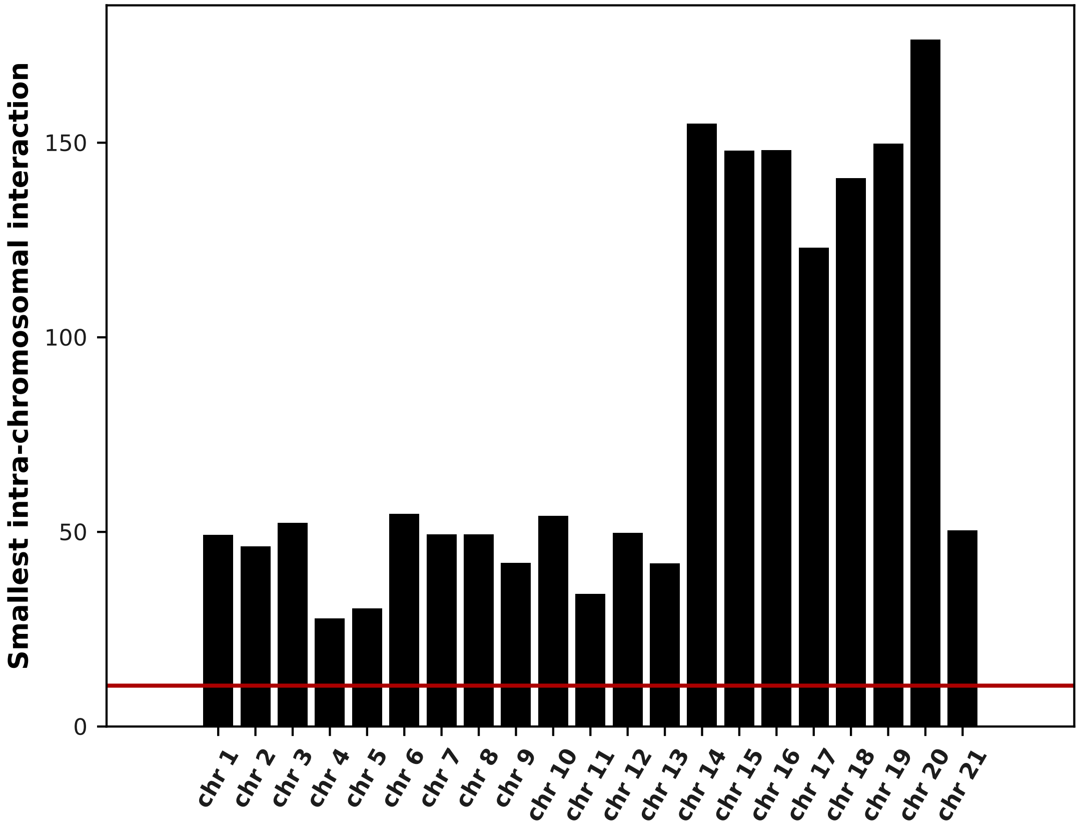

Figure S11: **transH versus cis.** Estimated smallest cis interaction for each chromosome (See Section 1.3). The red horizontal line corresponds to the estimated transH interaction.

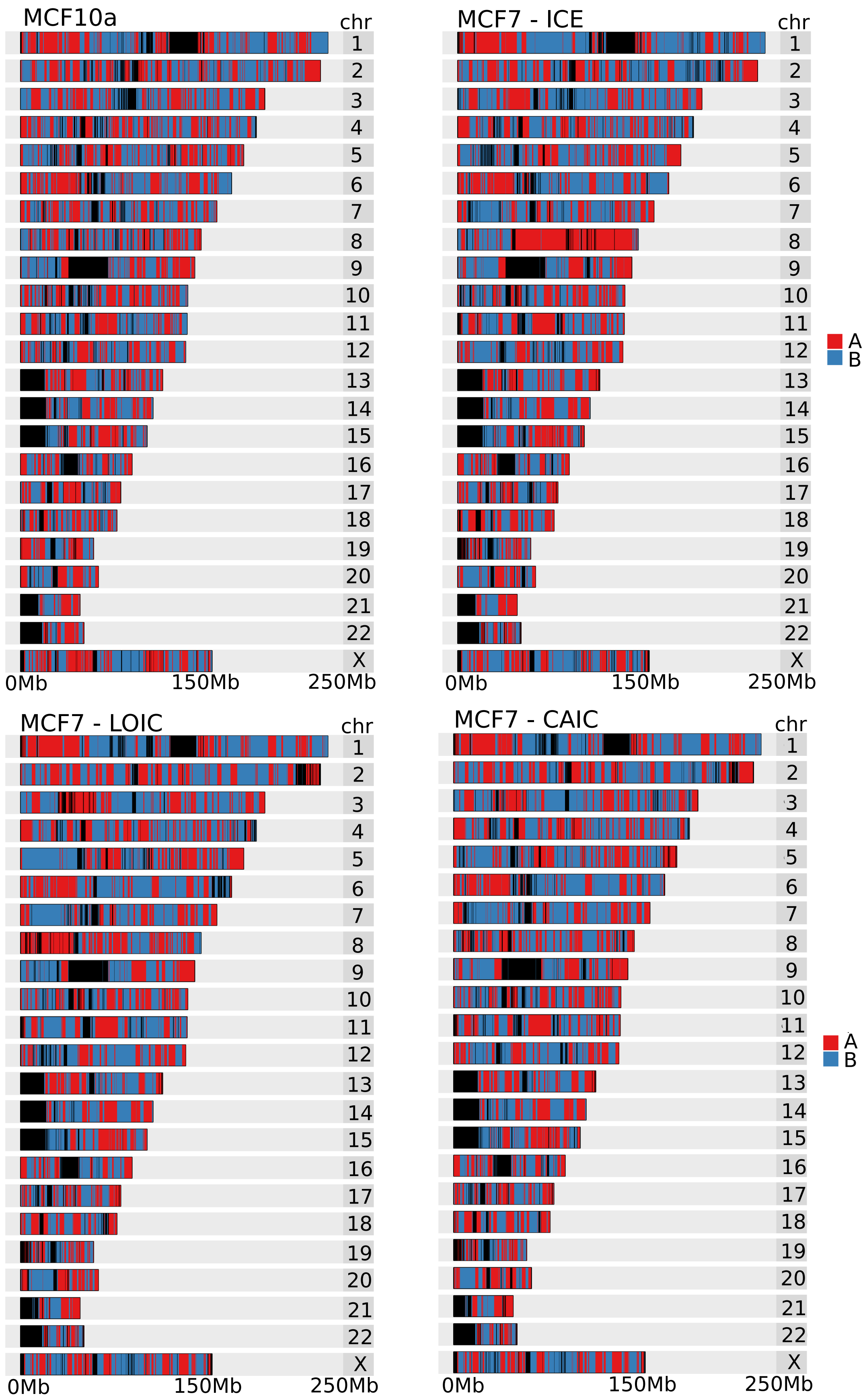

Figure S12: **MCF10A and MCF7 chromosome compartments.** Results of the compartment calling using MCF10A ICE normalized data and MCF7 data normalized with ICE, LOIC and CAIC methods. The active (A-type) and inactive (B-type) chromosome compartments are respectively represented in red and in blue.

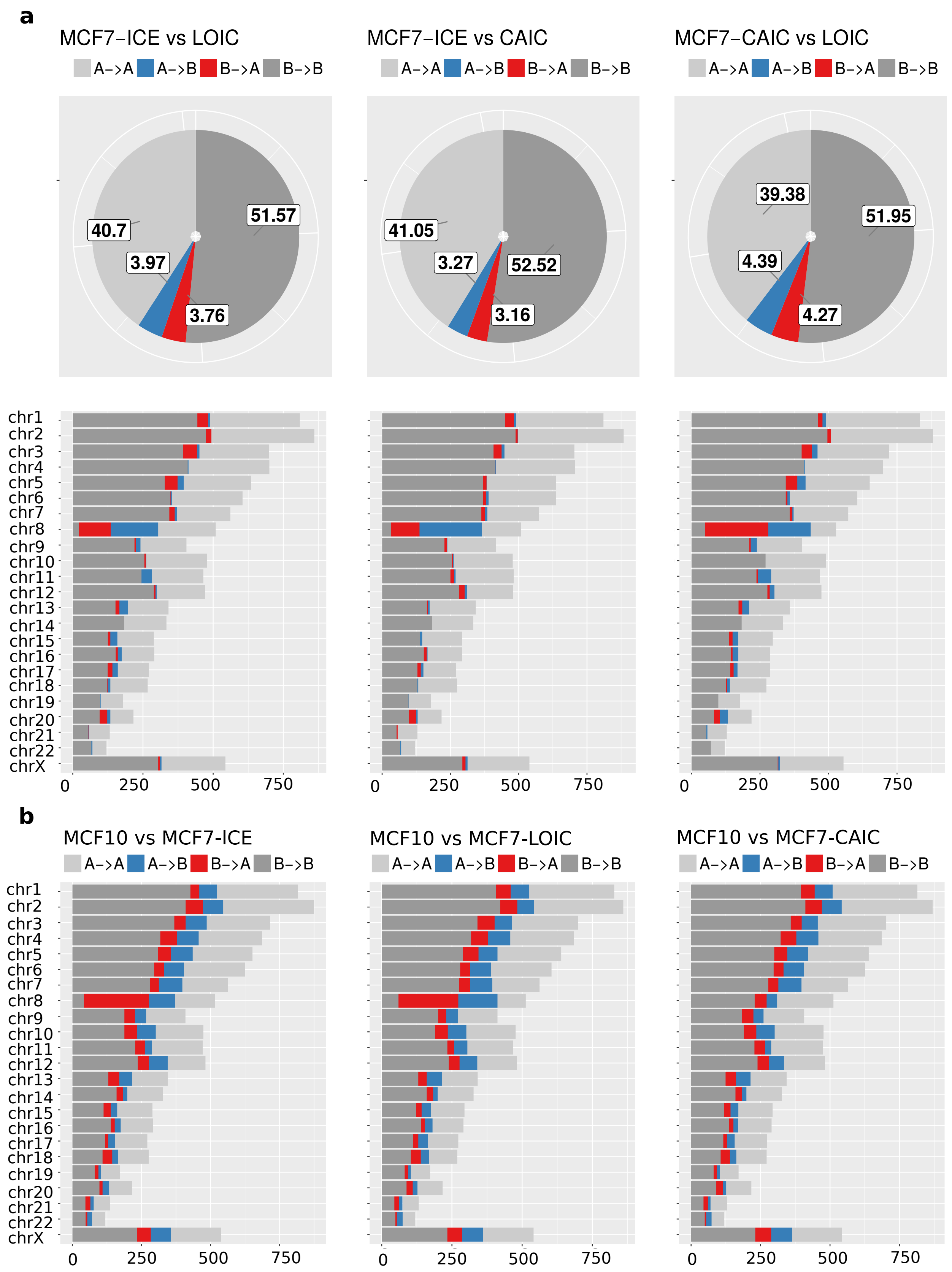

Figure S13: **Chromosome compartments switches between MCF7 normalized data and MCF10A data. a.** Overall and per-chromosome impact of the normalization method on MCF7 chromosome compartment calling. **b.** Impact of normalization on chromosome compartment switches between MCF10A and MCF7.
